# Revealing the Complexity of a Monogenic Disease: Rett Syndrome Exome Sequencing

**DOI:** 10.1371/journal.pone.0056599

**Published:** 2013-02-28

**Authors:** Elisa Grillo, Caterina Lo Rizzo, Laura Bianciardi, Veronica Bizzarri, Margherita Baldassarri, Ottavia Spiga, Simone Furini, Claudio De Felice, Cinzia Signorini, Silvia Leoncini, Alessandra Pecorelli, Lucia Ciccoli, Maria Antonietta Mencarelli, Joussef Hayek, Ilaria Meloni, Francesca Ariani, Francesca Mari, Alessandra Renieri

**Affiliations:** 1 Medical Genetics, University of Siena, Siena, Italy; 2 Genetica Medica, Azienda Ospedaliera Universitaria Senese, Siena, Italy; 3 Biochemistry and Molecular Biology, University of Siena, Siena, Italy; 4 Department of Surgery and Bioengineering University of Siena, Siena, Italy; 5 Neonatal Intensive Care Unit University Hospital Azienda Ospedaliera Universitaria Senese (AOUS) of Siena, Siena, Italy; 6 Department of Molecular and Developmental Medicine, University of Siena, Siena, Italy; 7 Child Neuropsychiatry Unit, University Hospital, AOUS, Siena, Italy; University of Bonn, Institut of experimental hematology and transfusion medicine, Germany

## Abstract

Rett syndrome (OMIM#312750) is a monogenic disorder that may manifest as a large variety of phenotypes ranging from very severe to mild disease. Since there is a weak correlation between the mutation type in the Xq28 disease-gene *MECP2*/X-inactivation status and phenotypic variability, we used this disease as a model to unveil the complex nature of a monogenic disorder. Whole exome sequencing was used to analyze the functional portion of the genome of two pairs of sisters with Rett syndrome. Although each pair of sisters had the same *MECP2 (*OMIM*300005) mutation and balanced X-inactivation, one individual from each pair could not speak or walk, and had a profound intellectual deficit (classical Rett syndrome), while the other individual could speak and walk, and had a moderate intellectual disability (Zappella variant). In addition to the *MECP2* mutation, each patient has a group of variants predicted to impair protein function. The classical Rett girls, but not their milder affected sisters, have an enrichment of variants in genes related to oxidative stress, muscle impairment and intellectual disability and/or autism. On the other hand, a subgroup of variants related to modulation of immune system, exclusive to the Zappella Rett patients are driving toward a milder phenotype. We demonstrate that genome analysis has the potential to identify genetic modifiers of Rett syndrome, providing insight into disease pathophysiology. Combinations of mutations that affect speaking, walking and intellectual capabilities may represent targets for new therapeutic approaches. Most importantly, we demonstrated that monogenic diseases may be more complex than previously thought.

## Introduction

The first publication of the catalogue of all known genes and genetic disorders, Mendelian Inheritance in Man (MIM), in 1966, fostered the idea that “rare diseases” were monogenic arising from single or double mutational events in one of the 29,000 genes of the human genome. On the contrary, “common diseases” are thought to be complex deriving from interactions between environmental factors and multiple mutational events in several genes, as well as epigenetic modifications. Incomplete penetrance, when individuals fail to express a trait, even when they have the trait-allele, and expression variability, wherein traits are expressed to different degrees among individuals with the same alleles, may suggest that also supposedly monogenic diseases are more complex than previously thought.

Rett syndrome (RTT) is a genetic neurodevelopmental disorder that is characterized by regression especially in the areas of language and motor abilities. [1] Studies have implicated *de novo* mutations of the methl-CpG-binding protein 2 (MeCP2) gene on the X chromosome in RTT. [2] RTT has a wide clinical spectrum. [1] Among the several hundred RTT sporadic patients that we have studied we encountered two rare familial cases consisting of pairs of sisters with RTT that are phenotypically discordant. That is, individuals in each pair of sisters demonstrate extremes of the RTT spectrum: classical RTT and Zappella RTT variant (Z-RTT). [3].

One factor that can modulate X-linked disorders is X chromosome inactivation (XCI) status. [4] However, all four mentioned individuals have a balanced XCI, indicating that other factors beyond XCI may contribute to the phenotypic outcome. [3], [5], [6] Thus, these pairs of sisters represent the ideal model to test the molecular basis of expression variability using an exome sequencing approach.

## Materials and Methods

### Patients

**Figure 1:**
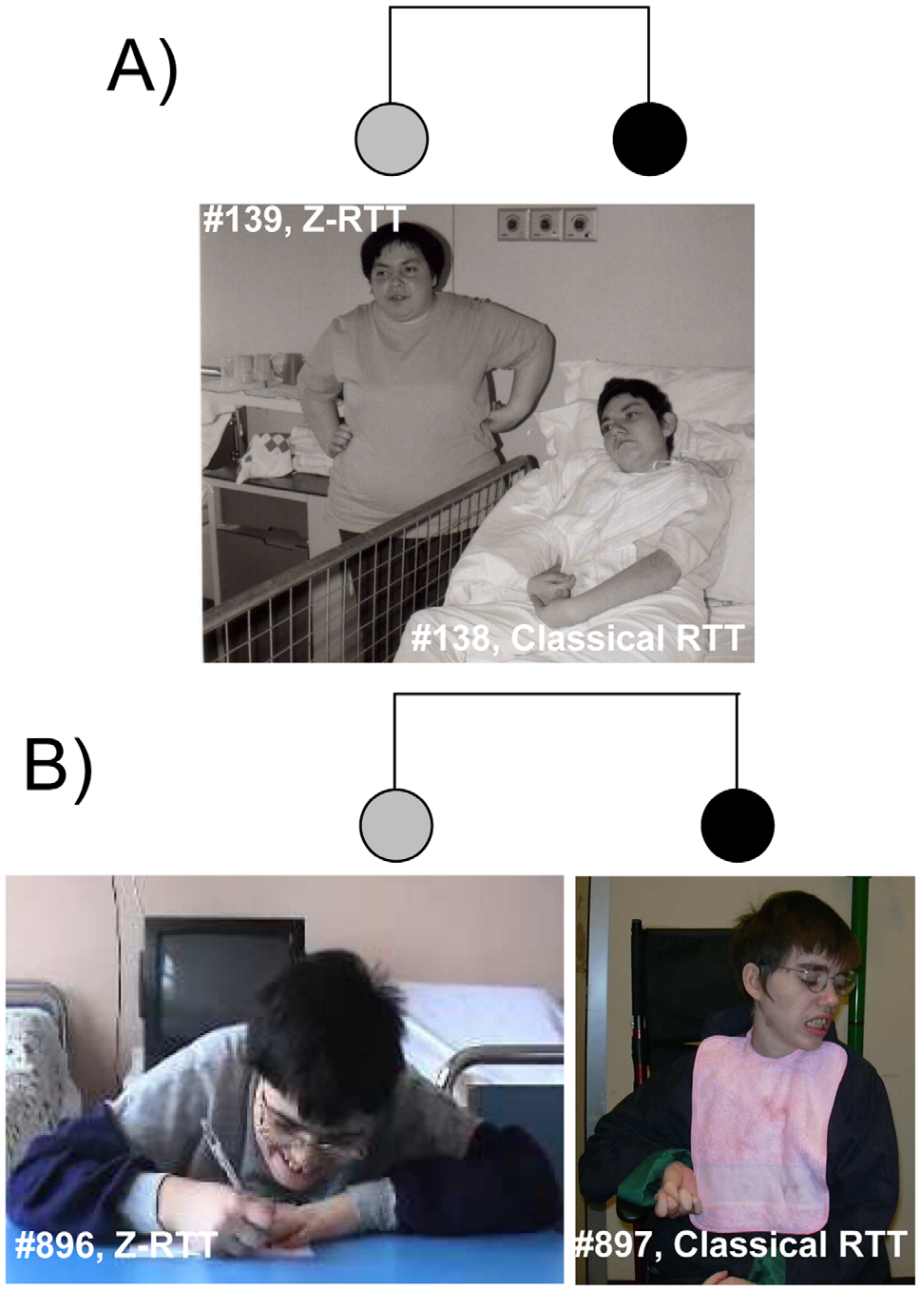
Patient photographs and pedigree. In the pedigrees the two sisters couples are represented by grey circles (milder variant = Zappella Rett variant (Z-RTT)) and black circles (more severe phenotype = classical Rett (RTT)). Panel a) Sisters #139 and #138 at the age of 28 and 19, respectively, and pedigree. Presently, patient #139 is 40 years old and is still able to speak in short phrases. Although late stage RTT-associated motor deterioration began 10 years ago, she is still ambulatory. Her phenotype was previously described. [3], [35] Her sister, patient #138, is 29 years old and has never been able to walk unassisted. Ten years ago she developed spastic tetraplegia with contractures that are still present and are further deteriorating. Panel b) Sisters #896 and #897 at the age of 32 and 26, respectively, and pedigree. Presently, patient #896 is 39 year-old and is still able to walk and to speak in short phrases. She has a friendly behavior and was extremely cooperative during examinations. Her somatic parameters is in the mean range (Occipital-Frontal Circumference (OFC): 54.5 cm, 50–75^th^ percentile; height 162 cm, 25–50^th^ percentile; weight 63 Kg, Body Mass Index (BMI) = 24), she has a severe kyphosis and mild pes planus. She has no hand stereotypes and possesses good manual abilities, being able to make simple drawings, eat independently, dress and wash herself. She has never had epilepsy, gastroesophageal reflux, breathing disorders and cold extremities. She has bruxism and a high pain threshold. Her 34 year-old sister (patient #897) shows spastic tetraplegia with severe contractures and hyperventilation. She shows somatic hypoevolutism (OFC 51,5 cm, <3rd percentile; height 150 cm, <3rd percentile; weight 29 Kg, BMI = 13), lordosis, and mild pes planus. She has constant hand stereotypes (pill counting and hand-mouthing), sialorrhea, bruxism, epilepsy that was not controlled by therapy, and cold extremities. She has never been able to speak.

Two pairs of sisters with discordant phenotype were enrolled in the study (Fig. 1a and 1b). Siblings #138 (classical RTT) and #139 (Z-RTT) possessed the same mutation in *MECP2*, c.1157del32, and showed a balanced XCI. The mutation was inherited from their unaffected mother, who had a completely skewed XCI. [3] Siblings #897 (classical RTT) and #896 (Z-RTT) had an apparently *de novo MECP2* deletion including exon 3 and part of exon 4. [6] XCI status analysis in this couple of sister revealed balanced XCI in both. [6] The unrelated classical RTT individuals #138 and #897 could not speak and walk and had a profound intellectual deficit, while the Z-RTT individuals #139 and #896 could speak and walk and had a moderate intellectual disability (Z-RTT). We quantified the striking differences in somatic, neurodevelopmental, and neurovegetative features between the sisters using a previously described scoring system (score from 0- mildest end to 40- most severe end; mean classical RTT score of 27.5±5.3 and mean Z-RTT score of 13.8±5.9; a threshold of 20 divided classical RTT from Z-RTT). [7] According to this scoring system the classical RTT girls had a clinical score of 30 (#138) and 33 (#897), which lies within the range of scores for the most severe RTT outcomes. Conversely, the Z-RTT girls had a score of 10 (#139) and 7 (#896) indicating a milder, high functioning form of RTT (Table 1). [7] This study was approved by the institutional review board of the University of Siena (Siena, Italy). The parents of the patients have given written informed consent, as outlined in the PLOS consent form, to publication of their photograph. Participation in the study did not alter the standard of care.

**Table 1:** Clinical features of the two couples of RTT sisters.

**ITEM**	**RTT sister pair 1**		**RTT sister pair 2**	
	**Patient #138**	**Patient #139**	**Patient #896**	**Patient #897**
**Age of assessment**	24 y	33 y	39 y 8 m	34 y
**Head (cm)**	2-Microcephaly	0-No deceleration	0-No deceleration	2-Microcephaly
**Weight (kg)**	2-Below 3^rd^ percentile	0-Above 25^th^ percentile	0-Above 25^th^ percentile	2-Below 3^rd^ percentile
**Height (cm)**	0-Above 25^th^ percentile	2-Below 5^th^ percentile	0-Above 25^th^ percentile	2-Below 5^th^ percentile
**Age of regression**	2-Before 18 months	1-Before 18 months	0-After 3 years	0-After 3 years
**Hand stereotypy**	2-Dominating or constant	1-Mild or intermittent	0-None	2-Dominating or costant
**Voluntary hand use**	2-None	0-Quite good hand use	0-Quite good hand use	2-None
**Sitting**	0-Sitting unsupported atage of 5	0-Sitting unsupported atage of 5	0-Sitting unsupported atage of 5	1-Loss of ability to sit
**Walking**	2-Never learned to walk	0-Walking unsupported atage of 5	0-Walking unsupported atage of 5	1-Loss of ability to walk
**Age of walk**	0-Before 18 months	0-Before 18 months	1-After and equal to18 months	1-After and equal to 18 months
**Speech**	2-Never spoken	0-More than 10 words atage of 5	0-More than 10 words atage of 5	2-Never spoken
**Age of increasing words**	2-Never	0-Before 6 years	1-After 6 years	2-Never
**Level of speech**	2-Absent	0-Phrases	0-Phrases	2-Absent
**Level of phrases**	2-Absent	1-Simple phrases	1-Simple phrases	2-Absent
**Epilepsy**	1-Controlled by therapy	0-No epilepsy at age of 5	0-No epilepsy at age of 5	2-Barely or not controlledby therapy
**Gastrointestinal disturbances**	2-Severe	0-Absent	0-Absent	0-Absent
**Breathing disorders**	2-Severe	0-Absent	0-Absent	2-Severe
**Cold extremities**	1-Mild	1-Mild	0-Absent	2-Severe
**Sphincter control**	1-Partial	0-Complete	0-Complete	2-Absent
**Genu valgu/Pes planus**	1-Mild	1-Mild	1-Mild	2-Severe
**Kyphosis**	0-Absent	1-Partial	2-Severe	0-Absent
**Scoliosis**	0-Absent	1-Mild	0-Absent	0-Absent
**Intellectual disability**	2-Non measurable: IQ<20	1-Severe IQ: 20–40	1-Severe IQ: 20–40	2-Non measurable: IQ<20
**TOTAL SCORE**	30	10	7	33

### Exome Sequencing and Data Analysis

Whole exome sequencing (WES) was performed using the Illumina platform in all 4 individuals (Methods S1 in File S1). Data were filtered against dbSNP132 and control populations (1000 Genomes Project Consortium; http://www.1000genomes.org/data). A further filtering was performedto retrieve only variants potentially altering protein function, according to predictive tools, i.e. truncating, splice site variants, and missense mutations probably alter protein function (Methods S1 in File S1).

## Results

The clinical and genetic data of the two pairs of RTT sisters are summarized in Table 1 and Fig. 1. Exome sequencing of 4 RTT subjects, after filtering against dbSNP132 and control populations (1000 Genomes Project Consortium; http://www.1000genomes.org/data), revealed that in addition to the *MECP2* mutation, each patient had about 2500 variants, 330 of which exonic and splicing changes. Using a combination of prediction tools, 82 variants per patient were predicted to potentially impair protein function (Tables S1–S3 in File S1). None of them were shared by the four individuals. The variants were grouped on the basis of the following criteria: i) exclusive to classical RTT girls (Table S1 in File S1); ii) exclusive to Z-RTT girls (Table S2 in File S1); iii) shared by two or three individuals with discordant phenotype (Table S3 in File S1).

**Figure 2:**
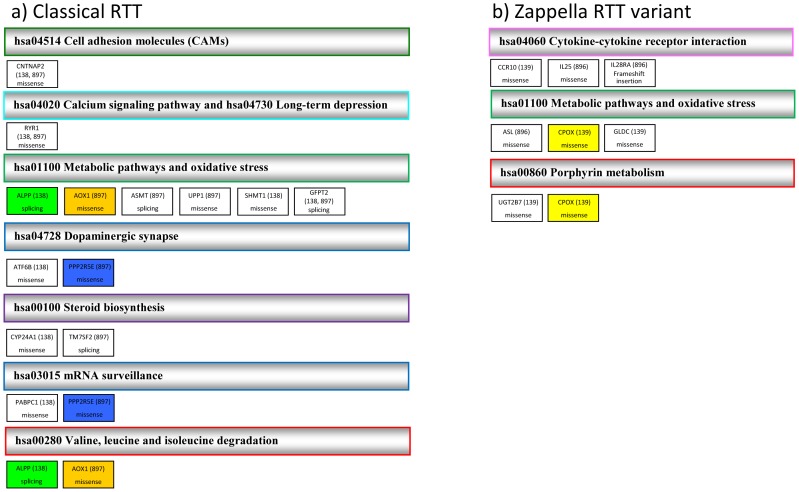
Relevant pathways of altered genes in classical Rett (a) and Zappella Rett variant girls (b). Only pathways in which at least two altered genes were included, or where one gene was mutated in either both classical Rett (RTT) (a) or both Zappella Rett variant (Z-RTT) (b) patients have been included. Genes that are involved in only one pathway are in white. Genes that are involved in more than one pathway are indicated with the same color. For each pathway the code assigned in the Kyoto Encyclopedia of Genes and Genomes (KEGG) database is indicated (see File S1). For each gene the mutation type is indicated.

The first group includes 112 variants belonging to 108 genes (Table S1 in File S1). Three genes, *CNTNAP2* (OMIM*604569), *GFPT2 (*OMIM*603865) and *RYR1* (OMIM*180901) had variations predicted to impair the protein function in both the unrelated classical RTT girls. These genes are involved in cell adhesion, oxidative stress and calcium signaling. Each classical RTT patient has in addition about 50 mutated genes among which we selected 21 potentially relevant genes through a meticulous analysis of the literature on Pubmed and taking into account if the genes where listed in OMIM and known to be associated with a neurological or neuromuscular phenotype (10 genes) (Table 2) and if the related protein was involved in a particular pathway (13 genes, 2 of which were already selected using the above mentioned criteria) (Fig. 2a). Interestingly, the two classical RTT patients shared alterations in pathways of steroid biosynthesis, dopaminergic synapses, mRNA surveillance and purine metabolism (Fig. 2a). Additional genes are associated with muscle impairment and intellectual disability and/or autism (Table 2).

**Table 2:** Variations predicted to impair protein function in disease/susceptibility genes related to muscle and brain.

Gene	Mutation type	Genotype(patient #)	Trait-related molecular mechanism	Susceptibility to	Associated disease (Inheritance)
**Classic RTT**					
*ABCA13*	missense	Heterozygous (897)	Unknown	Schizophrenia, bipolar disorder, depression	/
*AP4M1*	splicing	Heterozygous (138)	Neuroaxonal damage and glutamatereceptor abnormality	/	Spastic paraplegia and severe mental retardation (AR)
*ATRN*	missense	Heterozygous (138)	Causes obesity by mimickingagouti-related protein	/	/
*CNKSR2*	missense	Heterozygous (897)	Unknown	/	Non-Syndromic Intellectual Disability (XLR)
*CNTNAP2*	missense	Heterozygous(138/897)	/	Autism susceptibility 15	Pitt-Hopkins like syndrome 1 (AR), Cortical dysplasia-focal epilepsy syndrome (AR)
*DIRAS2*	missense	Heterozygous (897)	Unknown	Attention deficit/hyperactivity disorder (ADHD)	/
*KIAA0564*	frameshiftdeletion	Heterozygous (897)	Unknown	Autism	/
*KIF7*	missense	Heterozygous (138)	Regulation of GLI transcription factorsin SHH signaling pathway	/	Acrocallosal syndrome (AR)
					Hydrolethalus syndrome 2 (AR)
					Joubert syndrome 12 (AR)
*RYR1*	missense	Heterozygous(138/897)	Calcium signaling determiningcontraction of skeletal muscle	Malignant hyperthermia	Central core disease (AD and AR), Minicore myopathy with external ophthalmoplegia (AR), Neuromuscular disease, congenital, with uniform type 1 fiber (AD)
*TTC3*	missense	Heterozygous (138)	Inhibition of neuronal differentiation	Down syndrome	/
**Zappella variant**					
*ANK3*	missense	Heterozygous (139)	Synapse formation	Bipolar disorder	/
*ASL*	missense	Heterozygous (896)	Detoxification of ammonia via theurea cycle	/	Argininosuccinic aciduria (AR)
*COG7*	missense	Heterozygous (139)	Intracellular transport and glycoprotein modification	/	Congenital disorder of glycosylation, type II (AR)
*CPOX*	missense	Heterozygous (139)	Heme biosynthetic pathway	/	Coproporphyria (AD)
*GLDC*	missense	Heterozygous (139)	Degradation of glycine which has a neurotransmitter role	/	Glycine encephalopathy (AR)

The second group includes 80 variants/genes (Table S2 in File S1), none of them shared by both the unrelated Z-RTT girls. On the basis of shared pathway or disease association we selected an additional 9 genes using the same criteria described for classical RTT patients. Seven genes were selected on the basis of shared pathway and, interestingly, a subset of these genes are related to interleukine and chemokine receptors and, thus, may modulate immune responses (Fig. 2b). Additional 5 genes were associated with bipolar or metabolic disorders (Table 2). Three of them were already selected on the basis of shared pathway.

The third group of genes includes 64 variants in 62 genes that were shared by classical RTT and Z-RTT (Table S3 in File S1). Among them, 46 were mutated in either one pair of discordant sisters or the other.

**Figure 3:**
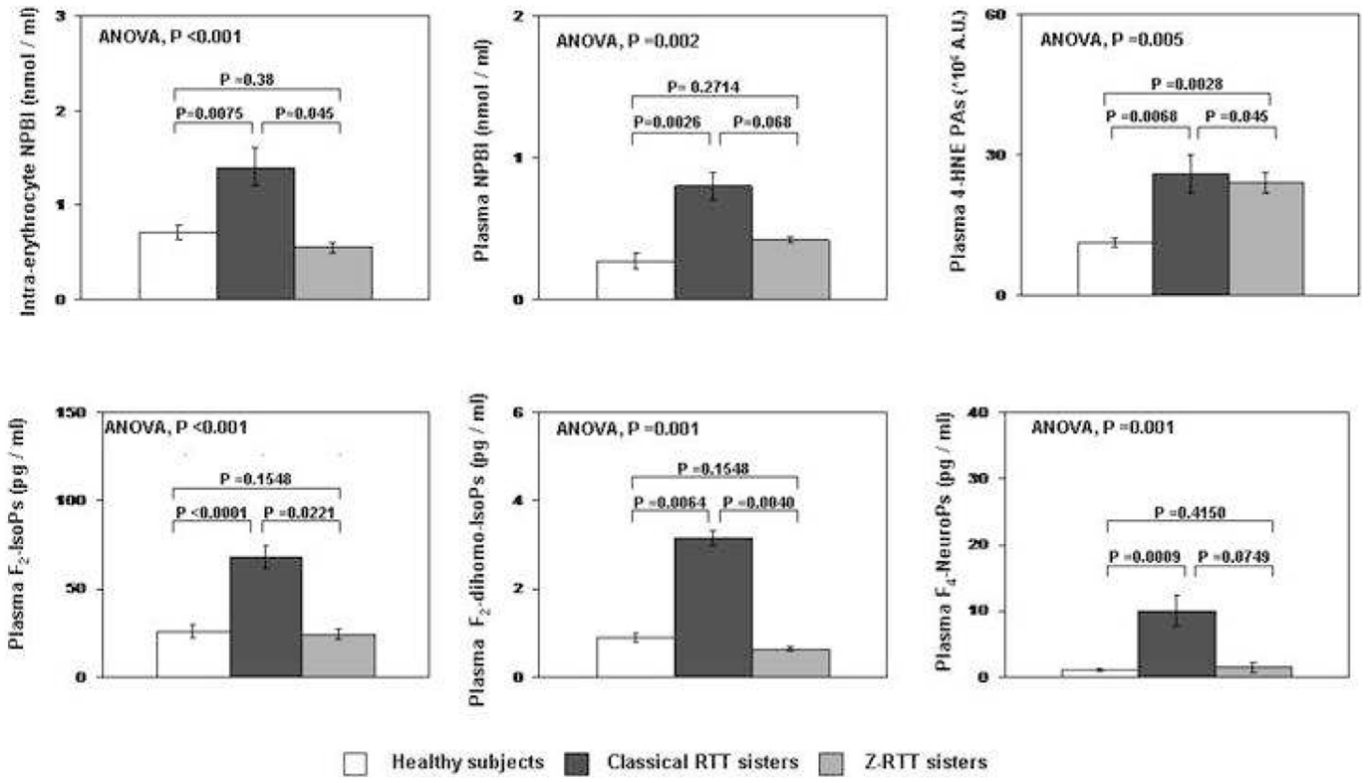
Comparison of oxidative stress markers in classical Rett versus Zappella Rett variant. In classical Rett (RTT) patients (N = 2), all the examined oxidative stress (OS) markers were significantly increased compared to healthy controls (N = 15, all females, mean age 36.5±4.2), whereas Zappella Rett variant (Z-RTT) patients (N = 2) behave as controls subjects except for plasma 4-HNE-PAs. Intra-erythrocyte and plasma non-protein bound iron (NPBI) are markers of hypoxia with hemoglobin oxidation and subsequent heme iron release. Plasma 4-HNE PAs is a marker of protein oxidation due to aldehyde binding from lipid peroxidation sources. F(2)-isoprostanes (F_2_-IsoPs) are the end-products of arachidonic acid oxidation, a polyunsaturated fatty acid that is abundant in both brain grey and white matter. F(2)-dihomo-isoprostanes (F_2_-dihomo-IsoPs) derive from oxidation of adrenic acid, a fatty acid abundant in white matter, specifically myelin. F(4)-neuroprostanes (F_4_-NeuroPs) are the end-products of docosahexanoic acid, abundant in neuronal membranes. Statistical differences were evaluated using Mann-Whitney sum rank test, Kruskal-Wallis analysis of variance (ANOVA) Two-tailed P-values are shown. Values are expressed as means ± standard error means (SEM); intra-erythrocyte NPBI is reported as nmol/ml erythrocytes suspension; plasma 4-HNE-PAs are expressed as arbitrary units (AU), while isoprostanes (IsoPs) are expressed as pg/ml.

Given the difference in the number of metabolic pathway genes related to oxidative stress (OS) in classical versus Z-RTT patients, we decided to test whether there was a difference in the OS phenotype. Interestingly, for five out of six OS markers (non-protein bound iron (NPBI), F(2)-dihomo-isoprostanes (F_2_-dihomo-IsoPs), F(3)-isoprostanes, F(4)-neuroprostanes (F_4_-NeuroPs), and F(2)-isoprostanes (F_2_-IsoPs)) there was not a statistically significant difference between Z-RTT and controls, while in classical RTT OS markers were significantly increased (Fig. 3).

## Discussion

RTT syndrome is usually due to *de novo* mutations in the *MECP2* gene. [2] Therefore, the vast majority of cases are sporadic. The two exceptional familial cases described here represent an ideal model to identify genetic modifiers underlying expression variability as in each couple there are two subjects manifesting both ends of the phenotype (Table 1 and Fig. 1), and since each couple will be enriched of identical variations facilitating the selection of those not shared.

The most important finding of this study is that it demonstrates that it is possible to use WES to gain insight into expression variability in a monogenic disease such as RTT. We demonstrated that each RTT subject had multiple mutations that may lead to functional variants. Potentially, all the mutations have a role in clinical manifestation and, despite our limited current knowledge about the function of genes, we have defined a subset that may cooperate to exacerbate (Table S1 in File S1) or ameliorate (Table S2 in File S1) the final clinical outcome (Fig. 1).

Both patients with classical RTT had different heterozygous missense mutations in the *RYR1* gene, a regulator of Ca^2+^ release, which is responsible for a number of clinical conditions, including a mild form of myopathy (Table S2 in File S1 and Fig. 2 and Table 2). The *RYR1* gene encodes the skeletal muscle ryanodine receptor, which serves as a calcium release channel of the sarcoplasmic reticulum, as well as being a bridging structure connecting the sarcoplasmic reticulum and transverse tubule. [8] *RYR1* mutations have been associated with several congenital neuromuscular disorders. The *RYR1* disrupting mutations identified in both classical RTT patients may contribute to the reduced muscle mass, weakness, and susceptibility to scoliosis exibited by classical RTT subjects but not in the Z-RTT patients.

Abrahams et al. noted that human *CNTNAP2* expression was enriched in circuits involved in higher cortical functions, including language. [9] *CNTNAP2* has been identified as an autism-susceptibility gene and recessive mutations cause Pitt-Hopkins-like syndrome 1 (OMIM#610042) and Cortical dysplasia-focal epilepsy syndrome (OMIM#610042 ). A *Cntnap2* knockout mouse model revealed neuronal migration abnormalities, a reduced number of interneurons, and abnormal neuronal network activity. [10] These mice also demonstrated deficits in the three core behavioral domains for Autism Spectrum Disorders (ASD), as well as hyperactivity and epileptic seizure. Treatment with the FDA-approved drug risperidone ameliorates the targeted repetitive behaviors in the mutant mice. These data demonstrated a functional role for *CNTNAP2* in brain development and provide a new tool for mechanistic and therapeutic research in ASDs. [10] In our study *CNTNAP2* mutations were observed in both classical subjects, but not in their Z-RTT sisters (Table S2 in File S1 and Fig. 2). Therefore, risperidone treatment may be a potentially strategy for the treatment of classical RTT patients.

We have previously reported a duplication in the 1q42.12 region in the Z-RTT patient #896 including *ENAH* (OMIM*609061). [5] This gene product localizes to cell-substrate adhesion sites and sites of dynamic actin assembly and disassembly participating in axonal outgrowth, dendrite morphology, synapse formation, and axon guidance. Although *ENAH* mutations are not listed in Tables S1–S3 in File S1, classical RTT patient #138 had a 4bp insertion (insAAAC) in the UTR3 region of the *ENAH* gene (position 225,675,743), at a site that is predicted to be conserved (Phylo P  = 0.52) (the entire list of mutations is available on request). This observation supports the role of *ENAH* in axon guidance and in the modulation of the RTT phenotype.

Our results indicated that the classical RTT subjects are likely to have a dysfunction in dopaminergic synapses due to functional variants in *ATF6B* (OMIM*600984) and *PPP2R5E* (OMIM*601647) genes encoding effectors of the postsynaptic cascade that follow binding of dopamine to D5 (OMIM+126453) receptor and D2 receptor (OMIM*126450), respectively (Table S1 in File S1 and Fig. 2a). This is in accordance with the finding that there is a reduction in the number and soma size of tyrosine hydroxylase-expressing neurons in a mouse model of RTT. [11] In this model, L-Dopa treatment ameliorated the motor deficits. [11] Interestingly, since dopamine D2-like partial agonists effectively treat respiratory disorders in the same mouse model, [12], functional alteration of genes involved in dopaminergic synapse, *ATF6B* and *PPP2R5E*, may exacerbate respiratory disorders typically observed in the 2 classical subjects. [13] Hyperventilation or breath holding was not noted in the two Z-RTT girls at any examination. Our analysis suggests that classical RTT patients may benefit from L-Dopa treatment more than their Z-RTT counterparts.

Both classical RTT subjects also likely had a partial block in the squalene catabolism, because of the presence of heterozygous mutations in *CYP24A1* (OMIM*126065) or *TM7SF2* (OMIM*603414), which encode the proteins CP24A and ERG24, respectively. CP24A metabolizes the step from the active form of vitamin D, calcitriol, to the inactive derivative calcitriol. Disruptive mutations in this enzyme may cause an increase in levels of calcitriol, cholesterol, and squalene. ERG24 catalyzes the step from 4-4dimethyl-cholesta-8,14,24-trienol to 14-demethylanosterol and mutations in this enzyme may also cause squalene accumulation. Very recently, it has been demonstrated that a mutation in squalene synthesis was found by randomly mutating a second genomic site in Mecp2-mutant mice, which was able to increase life span and decrease other RTT-like symptoms (Communication by Justice M, at 7th World Congress on Rett Syndrome, New Orleans, 2012). The same authors demonstrated that Mecp2 null mice develop non fatty acid liver storage disease (NAFLD), which is likely due to the link between Mecp2 and histone deacetylase-3 (*Hdac-3*, OMIM*605166) (Communication by Ebert D, at 7th World Congress on Rett Syndrome, New Orleans, 2012). Indeed, a recent report indicated that liver deletion of *Hdac-3* causes a metabolic syndrome and increases enzymes involved in cholesterol and lipid synthesis. [14], [15].

In the classical RTT subjects we observed mutations in the *TM7SF2* and *CYP24A1* genes, the gene products of which are part of a steroid cascade downstream from squalene epoxidase. Such mutations may have resulted in a partial block in the squalene metabolic pathway that, in concert with the *MECP2* haploinsufficiency, may have contributed to squalene accumulation. Together, these data support a possible role of modifier genes in cholesterol biosynthesis in RTT, and open the possibility to treatment of the patients with anti-cholesterolemic agents. Statins are a widely used and approved drugs and using specific outcome measures one can investigate whether this treatment may be effective in reducing some of the clinical outcomes of classical RTT patients.

Evidence of enhanced OS and lipid peroxidation has been reported in patients with RTT.[16]-[18] Furthermore, studies performed on hippocampus of the murine RTT model, mentioned above, showed increased oxidative burden, changes in mitochondrial function, and a more sensitive response to oxidative challenge. [19] The molecular mechanisms linking the *MECP2* gene mutation to the subsequent OS derangement are unknown to date. Recently, partial rescue of some of the neurological defects in RTT by ω-3 polyunsaturated fatty acids (PUFAs) has been reported. [20] In support of this, we identified in classical RTT subjects variants predicted to impair protein function in several genes involved in OS. *GFPT2*, which exhibited the same splice site mutation in both classical RTT girls, exerts a protective effect against H_2_O_2_ toxicity in neuronal HT-22 cells. [21] *AOX1*(OMIM*602841), catalyzes the formation of superoxide and is expressed in the ventral horn of the spinal cord, primarily in the glial cells. [22] Lastly, we identified mutations in *ASMT* (OMIM*300162), the gene product of which is involved in the synthesis of melatonin, a potent antioxidant (Fig. 2). As well as the genes reported in Fig. 2, we identified variations in other genes related to OS. These included *KCNJ14* (OMIM*603953)**,** a potassium channel whose expression is modified after oxidant exposure; *RICTOR* (OMIM*609022), a component of mTOR complex 2 whose expression is regulated by Sirtuin1, whose deficiency caused hepatic glucose overproduction, chronic hyperglycemia, and increased reactive oxygen species (ROS) production; and *ATF6B*, involved in the unfolded Protein Response pathway; and *RYR1* itself, as in *RYR1*related miopathies oxidant activity, the presence of OS markers and excessive production of oxidant by mithocondria has been shown. [23]–[25].

Using NPBI, 4HNE-PAs, and several isoprostanes (IsoPs) families as markers of redox derangement and lipid peroxidation, we confirmed our previous data demonstrating that OS is present in the classical RTT patients, while Z-RTT cases are more similar to controls (Fig.3). [26], [27].

The major novelty of the oxidative findings reported in the present study is that RTT patients with identical *MECP2* mutation, as our two pairs of sisters, can exhibit a different pattern of OS markers according to their clinical phenotype (i.e., concordant genotype with discordant phenotype). While confirming the co-existence of a significantly increased pro-oxidant status in genetically unrelated classical RTT subjects, the present data suggest that the redox alteration observed in RTT is likely to be modulated by genetic modifier factors, yet to be clarified. Earliest markers of hypoxia (NPBI), as well as those markers indicating general (F_2_-IsoPs) brain oxidative damage and specific grey (F_4_-NeuroPs) and white (F_2_-dihomo-IsoPs) matter injury, were elevated in classical RTT (Fig. 3). Interestingly, brain white matter damage has been previously reported in RTT; this supports the involvement of astrocytes in RTT, and their potential as therapeutic targets. [28], [29].

In the Zappella variant patients fewer affected genes are known to be involved in the OS pathway i.e. *ASL* (OMIM*608310), *CPOX* (OMIM*612732), and *GLDC* (OMIM*238300 ) (Fig. 2b). This observation is consistent with the results obtained measuring OS markers (Fig. 2). Z-RTT variants in fact behave as controls except for the levels of plasma 4-HNE PAs. This is an indicator of co-existing protein oxidation due to aldehyde binding in the presence of lipid peroxidation and its increase would suggest a milder chronic oxidative damage in Z-RTT possibly sparing the central nervous system (CNS).

Taken together, our results suggest that the genetic background underlying the *MECP2* mutation is strongly associated with OS in classical RTT patients and may contribute to a better understanding of the biological mechanisms for the observed benefits of PUFA supplementation in classical RTT patients. [30] On the opposite, our data seem to suggest that PUFA supplementation would be less efficient for Z-RTT patients. Our unpublished observations on a larger (n = 13) Z-RTT popupation (J. Hayek, unpublished data) appear to further support this speculation.

The role of the immune system in RTT has been demonstrated by the fact that transplantation of wild-type bone marrow restores wild-type microglia and arrests pathology in a mouse model of RTT. [29] It is, therefore, very interestingly that disrupting mutations in chemokines and chemokines receptors were found in Z-RTT patients, but not in the classical RTT patients. Chemokine receptor CCR10 (OMIM*600240) is known to be expressed in astrocytes. [31] There is evidence suggesting that selected chemokines can induce further chemokine synthesis in astrocytes, providing a mechanism to amplify inflammatory responses in CNS. [31] The *IL28RA* (OMIM*607404) gene has a frame-shift mutation that probably prevents its binding to members of the potent anti-inflammatory IL10 family cytokine (IL28A (OMIM*607401), IL28B (OMIM*607402) and IL29 (OMIM*607403)). A missense mutation in IL25 (IL17E, OMIM*605658), which belongs to the pro-inflammatory IL17 family of cytokines, likely leads to alterations in protein function. We hypothesize that this combination of mutations along with the *MECP2* disruption may modulate the immune system in a clinically favorable way. It is difficult to speculate on the exact role of this modulation, since the mechanism by which bone marrow transplantation exerts beneficial effects is unknown at present. Z-RTT patients may potentially have a more pronounced inflammatory response. It is also possible that Z-RTT subjects have a less efficient inflammatory response to internal or external (adjuvant of vaccines) stimuli. A more active response to such stimuli may worsen the CNS damage in classical RTT patients. Treating classical RTT with immunomodulators, such as IL-10 (OMIM*124092) would be an innovative strategy worthy of investigation.

It has been reported that the type of *MECP2* mutation and the X-inactivation status influence the clinical outcome of RTT. [4] However, in the current study each pair of sisters had the same *MECP2* mutation and XCI. [5] Thus, these two pairs of sisters represent an ideal model to test additional factors that modulate the expression variability.

It is well known that the genetic background of mouse models can influence phenotypic expression. The mouse model developed in 2001 successfully phenocopies a number of aspects of RTT, whereas previous models have failed in this attempt. [32], [33] Presently, it would be interesting to compare the genetic backgrounds of mice (employed in previous mouse RTT models) in which *MECP2* mutations do not produce the RTT phenotype with that of the current model. [32] In doing so, the contribution of alterations in the dopaminergic system or of the oxidative burden and mitochondrial dysfunction may be confirmed. [11], [19].

The study of familial cases of RTT offers the opportunity to identify the different molecular pathways involved in the expression of discordant phenotypes. Our data show that evaluating the degree of OS imbalance in patients with RTT may also be important in fully understanding the disease outcomes. OS status is known to be under the control of several transcription factors and, in turn, plays a major role in cell signaling and hence constitutes a potential phenotype modifier in RTT. [34].

Together, our data indicate that the final phenotype in RTT patients is likely the result of a combination of mutations in *MECP2*, X inactivation status, and 40–50 disrupting variants in other genes. Importantly, our study may have identified novel targets for personalized RTT pharmacological intervention.

## Supporting Information

File S1 **Supporting methods, tables, references.** Methods S1. Table S1, Variations predicted to impair protein function exclusive to classical RTT patients. Table S2, Variations predicted to impair protein function exclusive to Z-RTT patients. Table S3, Variations predicted to impair protein function in discordant RTT patients. References S1.
(DOC)
