# Supplementary material for: Revealing the Complexity of a Monogenic Disease: Rett Syndrome Exome Sequencing
Source: PLoS One. 2013 Feb 28;8(2):e56599. doi: 10.1371/journal.pone.0056599 (PMC3585308; doi:10.1371/journal.pone.0056599)
Supplement: File S1 — Supporting methods, tables, references. Methods S1. Table S1, Variations predicted to impair protein function exclusive to classical RTT patients. Table S2, Variations predicted to impair protein function exclusive to Z-RTT patients. Table S3, Variations predicted to impair protein function in discordant RTT patients. References S1. (DOC) [file pone.0056599.s001.doc]

**Methods S1**

Exome sequencing

“Whole exome sequencing” (Illumina platform) was used to perform an unbiased analysis of the functional portion of the genome in the subjects. All genomic DNA samples went through quality controls for DNA concentration and quality (PicoGreen and 1% agarose gel). Sample library and exome capture were performed using the TrueSeq Exome Illumina kit (Illumina, Inc. San Diego, CA). Post-Library Quality Controls were performed by Agilent 2100 Bioanalyzer. The clonal clusters were created on version 3 flowcells and the pair-end 2X100bp sequencing was performed on HiSeq2000, following the standard Illumina protocol.

More than 80% of the target sequences were covered by 10 or more reads and 59% of sequenced gene regions had more than 20 reads. After data analysis, we validated by Sanger sequencing the variations in candidate genes with a coverage <20 reads. In-house software was developed to analyze the exome sequencing data. The code was written in Python 2.7 and is freely available upon request. The program allows filtering for mutation type and allows a comparison of whole exome sequencing data among patients and controls. The system allows for the comparison of certain groups of subjects. For example, it is possible to compare all the patients of a test set versus a control set, or subgroups of patients with different phenotypic characteristics versus other subgroups (i.e. classical versus Z-RTT patients) or controls. In the current study, we used the program to retrieve exonic mutations (excluding synonymous) and splicing mutations for each patient. Secondly, we identified genes mutated in all 4 RTT subjects that were not mutated in controls, and genes mutated in all 4 RTT irrespective of controls. Finally, we created a list of (i) genes mutated only in one or both classical patients and not in Z-RTT subjects irrespective of controls, (ii) genes mutated in one or both Z-RTT variants and not in classical RTT irrespective of controls, and (iii) genes mutated in at least one classical and one Z-RTT patient irrespective of controls.

Pathway analysis.

The KEGG database (http://www.genome.jp/kegg-bin/show_organism?org=hsa),[1, 2] a resource that integrates genomic, chemical, and systemic functional information, was used to determine the human pathways in which the different genes were involved. We considered all pathways in which a particular gene is involved.

Oxidative stress markers

The level of oxidative stress (OS) imbalance was evaluated using a series of blood markers.[3] Blood was collected in heparinized tubes, and all manipulations were carried out within 2 hrs of collection. Blood samples were centrifuged at 2,400 X g for 15 min at room temperature. The platelet poor plasma and erythrocytes were obtained as described.[4] We quantified free F_2_-IsoPs, F_4_-NeuroPs, F_2_-dihomo-IsoProstanes (F_2_-dihomo-IsoPs), non protein-bound iron (NPBI), and 4-hydroxynonenal plasma protein adducts (4HNE-PAs) in the plasma. Intra-erythrocyte NPBI was also determined. In both plasma and erythrocytes, NPBI was determined as a desferrioxamine-iron complex by high-performance liquid chromatography, as previously reported.[5] F_2_-IsoPs, F_4_-NeuroPs, and F_2_-dihomo-IsoPs were determined by a gas chromatography/negative ion chemical ionization tandem mass spectrometry (GC/NICI-MS/MS) analysis after solid-phase extraction and derivatization steps.[4] 4HNE-PAs were examined by Western blot analysis.[6]

Data mining

We performed variation analysis with 3 prediction programs: SIFT, PolyPhen-2 and PhyloP.

SIFT (<http://sift.jcvi.org/www/SIFT_BLink_submit.html>) and PolyPhen-2 (http://genetics.bwh.harvard.edu/pph2/index.shtml) are tools that can predict the potential impact of an amino acid substitution on the structure and function of a human protein. The analyses producing categorical output that is tolerated/not tolerated for SIFT and benign/possibly damaging/probably damaging for PolyPhen-2. Furthermore, PolyPhen-2 gives a score between 0 and 1 (the higher the score, the more damaging the variation) and indicates a value for sensitivity and specificity of the result. Only variants not tolerated for SIFT and possibly/probably damaging by PolyPhen-2 were considered as functionally relevant. Variants with discordant results from the two programs were also retained when another variation with concordant results of pathogenicity was found in the same gene in another patient.

PhyloP (<http://genome.ucsc.edu/cgi-bin/hgGateway>) measures the phylogenetic conservation of a nucleotide at a specific position and provides a numerical value, ranging from -3.69 to +6.94. Positive scores are assigned to sites predicted to be conserved and negative scores to sites predicted to be fast evolving.

SIFT, PolyPhen-2 and PhyloP have been used for nonsynonymous variations. PhyloP alone has been used for in-frame insertions and deletions, stopgain and splicing variations. PhyloP score for in-frame insertions and deletions was calculated as the average score of all positions involved.

**Table S1. Variations predicted to impair protein function exclusive to classical RTT patients.**

| sample | chr | genomic position | genotype | gene | variation type | RefSeq | exon | nucleotide variation | protein variation | SIFT | PolyPhen-2 | | PhyloP |
| --- | --- | --- | --- | --- | --- | --- | --- | --- | --- | --- | --- | --- | --- |
| 897 | chr7 | 48318552 | hetero | ABCA13 | nonsynonymous SNV | NM_152701 | exon18 | c.A7761G | p.I2587M | not tolerated | possibly damaging | 0.697 (sensitivity: 0.86; specificity: 0.92) | -0.0544803 |
| 138 | chr2 | 233243796 | hetero | ALPP | splicing | NM_001632 | exon2 | c.T192C | p.D64D | n.a. | n.a. | n.a. | -3.04665 |
| 897 | chr2 | 201467043 | hetero | AOX1 | nonsynonymous SNV | NM_001159 | exon6 | c.T473C | p.I158T | not tolerated | possibly damaging | 0.578 (sensitivity: 0.88; specificity: 0.91) | 1.11456 |
| 138 | chr7 | 99703582 | hetero | AP4M1 | splicing | NM_004722 | exon12 | c.G930A | p.R310R | n.a. | n.a. | n.a. | 1.2928 |
| 897 | chr2 | 232135771 | hetero | ARMC9 | nonsynonymous SNV | NM_025139 | exon13 | c.C1196T | p.A399V | not tolerated | probably damaging | 1.000 (sensitivity: 0.00; specificity: 1.00) | 2.469 |
| 897 | chrX | 1748834 | hetero | ASMT | splicing | NM_004043 |  | c.562+2T>C |  | n.a. | n.a. | n.a. | 0.36251 |
| 138 | chr6 | 32084337 | hetero | ATF6B | nonsynonymous SNV | NM_001136153 | exon17 | c.A1793T | p.H598L | not tolerated | probably damaging | 1.000 (sensitivity: 0.00; specificity: 1.00) | 2.14094 |
| 897 | chr13 | 25275011 | hetero | ATP12A | nonsynonymous SNV | NM_001676 | exon13 | c.G1832A | p.R611Q | not tolerated | probably damaging | 1.000 (sensitivity: 0.00; specificity: 1.00) | 1.58514 |
| 897 | chr1 | 154316028 | homo | ATP8B2 | nonsynonymous SNV | NM_020452 | exon17 | c.C1841T | p.T614I | not tolerated | probably damaging | 1.000 (sensitivity: 0.00; specificity: 1.00) | 1,51754 |
| 138 | chr20 | 3565356 | hetero | ATRN | nonsynonymous SNV | NM_139322 | exon18 | c.T3013G | p.C1005G | not tolerated | probably damaging | 1.000 (sensitivity: 0.00; specificity: 1.00) | 2.16513 |
| 138 | chr17 | 65988139 | hetero | C17orf58 | nonsynonymous SNV | NM_181655 | exon3 | c.C184T | p.R62C | not tolerated | probably damaging | 0.999 (sensitivity: 0.14; specificity: 0.99) | 2.09908 |
| 897 | chr1 | 247712523 | hetero | C1orf150 | splicing | NM_145278 |  | c.29+1G>A |  | n.a. | n.a. | n.a. | 2.096 |
| 138 | chr20 | 3297387 | hetero | C20orf194 | nonsynonymous SNV | NM_001009984 | exon18 | c.C1522G | p.Q508E | n.d. | n.d. | n.d. | 1.01766 |
| 897 | chr20 | 20322489 | hetero | C20orf26 | nonsynonymous SNV | NM_015585 | exon26 | c.C3437T | p.P1146L | not tolerated | probably damaging | 0.981 (sensitivity: 0.75; specificity: 0.96) | 2.39507 |
| 897 | chr12 | 25343559 | hetero | CASC1 | nonsynonymous SNV | NM_018272 | exon2 | c.A10G | p.K4E | not tolerated | possibly damaging | 0.666 (sensitivity: **0.86**; specificity: **0.91**) | 0.313535 |
| 897 | chr17 | 20799056 | homo | CCDC144NL | nonsynonymous SNV | NM_001004306 | exon1 | c.T278A | p.V93D | not tolerated | possibly damaging | 0.711 (sensitivity: 0.86; specificity: 0.92) | 0.657921 |
| 897 | chr3 | 180334077 | hetero | CCDC39 | frameshift insertion | NM_181426 | exon19 | c.2660_2661insT | p.L887fs | n.a. | n.a. | n.a. | n.a. |
| 138 | chr6 | 31084805 | hetero | CDSN | nonsynonymous SNV | NM_001264 | exon2 | c.C587T | p.S196F | not tolerated | possibly damaging | 0.904 (sensitivity: 0.82; specificity: 0.94) | 0.260307 |
| 138 | chr1 | 86889769 | hetero | CLCA2 | splicing | NM_006536 |  | c.1-162T>G |  | n.a. | n.a. | n.a. | 0,905283 |
| 897 | chrX | 21450741 | hetero | CNKSR2 | nonsynonymous SNV | NM_001168649 | exon3 | c.G240T | p.L80F | tolerated | benign | 0.238 (sensitivity: 0.91; specificity: 0.88) | 0.722811 |
| 897 | chrX | 21450753 | hetero | CNKSR2 | nonsynonymous SNV | NM_001168649 | exon3 | c.T252A | p.N84K | not tolerated | probably damaging | 0.975 (sensitivity: 0.76; specificity: 0.96) | 1.52898 |
| 138 | chr7 | 147183121 | hetero | CNTNAP2 | nonsynonymous SNV | NM_014141 | exon11 | c.A1765C | p.T589P | not tolerated | probably damaging | 0.999 (sensitivity: 0.14; specificity: 0.99) | 2.22642 |
| 897 | chr7 | 147336347 | hetero | CNTNAP2 | nonsynonymous SNV | NM_014141 | exon13 | c.G2047A | p.E683K | tolerated | possibly damaging | 0.953 (sensitivy: 0.89; specificity: 0.95) | 2.873 |
| 897 | chr1 | 230795321 | hetero | COG2 | nonsynonymous SNV | NM_001145036 | exon2 | c.G184A | p.E62K | not tolerated | probably damaging | 1.000 (sensitivity: 0.00; specificity: 1.00) | 2.46256 |
| 138 | chr6 | 56044835 | hetero | COL21A1 | nonsynonymous SNV | NM_030820 | exon3 | c.G181T | p.V61F | not tolerated | probably damaging | 0.971 (sensitivity: 0.77; specificity: 0.96) | 0.990528 |
| 897 | chr1 | 86334218 | hetero | COL24A1 | nonsynonymous SNV | NM_152890 | exon37 | c.G3284A | p.G1095D | not tolerated | probably damaging | 1.000 (sensitivity: 0.00; specificity: 1.00) | 1.33851 |
| 897 | chr4 | 8621243 | homo | CPZ | nonsynonymous SNV | NM_001014448 | exon11 | c.C1447T | p.R483W | not tolerated | probably damaging | 0.985 (sensitivity: 0.74; specificity: 0.96) | 0.269827 |
| 897 | chr6 | 43190293 | hetero | CUL9 | nonsynonymous SNV | NM_015089 | exon37 | c.C6946T | p.R2316W | not tolerated | probably damaging | 0.978 (sensitivity: 0.76; specificity: 0.96) | 2.32916 |
| 138 | chr20 | 52789916 | hetero | CYP24A1 | nonsynonymous SNV | NM_000782 | exon1 | c.G203A | p.G68D | not tolerated | probably damaging | 1.000 (sensitivity: 0.00; specificity: 1.00) | 2.37419 |
| 138 | chr17 | 5365782 | hetero | DHX33 | nonsynonymous SNV | NM_001199699 | exon2 | c.G16A | p.A6T | not tolerated | probably damaging | 0.995 (sensitivity: 0.68; specificity: 0.97) | 2.60308 |
| 897 | chr9 | 93375599 | hetero | DIRAS2 | nonsynonymous SNV | NM_017594 | exon2 | c.C511T | p.R171C | not tolerated | probably damaging | 0.997 (sensitivity: 0.41; specificity: 0.98) | 2.884 |
| 897 | chr3 | 58194024 | hetero | DNASE1L3 | nonsynonymous SNV | NM_004944 | exon4 | c.C151T | p.R51C | not tolerated | probably damaging | 1.000 (sensitivity: 0.00; specificity: 1.00) | 2.622 |
| 138 | chr11 | 6532559 | hetero | DNHD1 | nonsynonymous SNV | NM_173589 | exon6 | c.A1292G | p.Y431C | not tolerated | probably damaging | 1.000 (sensitivity: 0.00; specificity: 1.00) | 2.09702 |
| 138 | chr6 | 116720448 | hetero | DSE | nonsynonymous SNV | NM_013352 | exon2 | c.T35A | p.F12Y | not tolerated | possibly damaging | 0.943 (sensitivity: 0.80; specificity: 0.95) | 1.16509 |
| 138 | chr3 | 184005700 | hetero | ECE2 | nonsynonymous SNV | NM_014693 | exon11 | c.G1693A | p.V565M | not tolerated | probably damaging | 1.000 (sensitivity: 0.00; specificity: 1.00) | 2.24843 |
| 138 | chr5 | 111500816 | hetero | EPB41L4A | splicing | NM_022140 |  | c.1933-2->TTTTA |  | n.a. | n.a. | n.a. | 2.12393 |
| 897 | chr8 | 139180195 | hetero | FAM135B | nonsynonymous SNV | NM_015912 | exon12 | c.G1201A | p.D401N | not tolerated | probably damaging | 1.000 (sensitivity: 0.00; specificity: 1.00) | 2.81281 |
| 138 | chr4 | 126412109 | hetero | FAT4 | nonsynonymous SNV | NM_024582 | exon17 | c.C14132T | p.T4711M | not tolerated | possibly damaging | 0.776 (sensitivity: 0.85; specificity: 0.92) | 1.02854 |
| 138 | chr3 | 46011166 | hetero | FYCO1 | splicing | NM_024513 |  | c.630+1G>A |  | n.a. | n.a. | n.a. | 2.734 |
| 897 | chr2 | 109116049 | hetero | GCC2 | nonsynonymous SNV | NM_181453 | exon22 | c.A4823G | p.E1608G | not tolerated | probably damaging | 1.000 (sensitivity: 0.00; specificity: 1.00) | 0.757803 |
| 138 | chr5 | 179765491 | hetero | GFPT2 | splicing | NM_005110 |  | c.115+2T>G |  | n.a. | n.a. | n.a. | 2.14875 |
| 897 | chr5 | 179765491 | hetero | GFPT2 | splicing | NM_005110 |  | c.115+2T>G |  | n.a. | n.a. | n.a. | 2.14875 |
| 897 | chr7 | 150439985 | hetero | GIMAP1-GIMAP5 | nonsynonymous SNV | NM_001199577 | exon6 | c.T1370G | p.V457G | not tolerated | probably damaging | 0.998 (sensitivity: 0.27; specificity: 0.99) | 1.72428 |
| 138 | chr15 | 89424908 | hetero | HAPLN3 | frameshift deletion | NM_178232 | exon3 | c.172delC | p.L58fs | n.a. | n.a. | n.a. | n.a. |
| 897 | chr10 | 14896155 | hetero | HSPA14 | nonsynonymous SNV | NM_016299 | exon9 | c.C766G | p.R256G | not tolerated | probably damaging | 1.000 (sensitivity: 0.00; specificity: 1.00) | 1.3493 |
| 897 | chr19 | 55967772 | hetero | ISOC2 | nonsynonymous SNV | NM_024710 | exon2 | c.C82T | p.R28C | not tolerated | probably damaging | 1.000 (sensitivity: 0.00; specificity: 1.00) | 2.074 |
| 138 | chr19 | 48967816 | hetero | KCNJ14 | nonsynonymous SNV | NM_013348 | exon3 | c.G1093A | p.E365K | not tolerated | possibly damaging | 0.899 (sensitivity: 0.82; specificity: 0.94) | 2.837 |
| 897 | chr13 | 42361607 | hetero | KIAA0564 | frameshift deletion | NM_015058 | exon18 | c.2132_2135delTCAG | p.711_712del | n.a. | n.a. | n.a. | n.a. |
| 138 | chr15 | 90191895 | hetero | KIF7 | nonsynonymous SNV | NM_198525 | exon5 | c.G1034A | p.R345H | not tolerated | probably damaging | 1.000 (sensitivity: 0.00; specificity: 1.00) | 1.97674 |
| 897 | chr3 | 47287699 | hetero | KIF9 | nonsynonymous SNV | NM_022342 | exon13 | c.G1277T | p.R426L | not tolerated | possibly damaging | 0.902 (sensitivity: 0.82; specificity: 0.94) | 1.4122 |
| 138 | chr19 | 51584912 | hetero | KLK14 | nonsynonymous SNV | NM_022046 | exon4 | c.C137T | p.T46M | not tolerated | probably damaging | 1.000 (sensitivity: 0.00; specificity: 1.00) | 0.214386 |
| 138 | chr17 | 39274194 | hetero | KRTAP4-11 | nonsynonymous SNV | NM_033059 | exon1 | c.G374A | p.C125Y | not tolerated | probably damaging | 1.000 (sensitivity: 0.00; specificity: 1.00) | 1.27817 |
| 897 | chr1 | 201356004 | hetero | LAD1 | frameshift deletion | NM_005558 | exon3 | c.483_484delCC | p.161_162del | n.a. | n.a. | n.a. | n.a. |
| 897 | chr6 | 161022077 | hetero | LPA | nonsynonymous SNV | NM_005577 | exon20 | c.G2999A | p.C1000Y | not tolerated | possibly damaging | 0.526 (sensitivity: 0.88; specificity: 0.90) | 1.322 |
| 897 | chr1 | 113659057 | hetero | LRIG2 | splicing | NM_014813 | exon16 | c.C2679T | p.D893D | n.a. | n.a. | n.a. | 0.48369 |
| 138 | chr4 | 110791416 | hetero | LRIT3 | stopgain SNV | NM_198506 | exon3 | c.G1376A | p.W459X | n.a. | n.a. | n.a. | 2.30139 |
| 897 | chr3 | 169514027 | hetero | LRRC34 | frameshift deletion | NM_153353 | exon8 | c.942delT | p.L314fs | n.a. | n.a. | n.a. | n.a. |
| 138 | chr6 | 90463812 | hetero | MDN1 | nonsynonymous SNV | NM_014611 | exon21 | c.C2954T | p.S985L | not tolerated | possibly damaging | 0.630 (sensitivity: 0.87; specificity: 0.91) | 2.54342 |
| 138 | chr19 | 36211374 | hetero | MLL4 | nonframeshift deletion | NM_014727 | exon3 | c.1126_1128delAAG | p.376_376del | n.a. | n.a. | n.a. | 0.504074 |
| 897 | chr5 | 7889387 | hetero | MTRR | splicing | NM_002454 | exon9 | c.C1326T | p.L442L | n.a. | n.a. | n.a. | -3.83437 |
| 897 | chr15 | 72338665 | hetero | MYO9A | nonsynonymous SNV | NM_006901 | exon2 | c.G240C | p.M80I | n.d. | n.d. | n.d. | 1.21094 |
| 897 | chr4 | 17846487 | hetero | NCAPG | splicing | NM_022346 |  | c.3048+1439A>C |  | n.a. | n.a. | n.a. | 0.065252 |
| 897 | chr17 | 29508805 | hetero | NF1 | splicing | NM_001042492 |  | c.730+2T>G |  | n.a. | n.a. | n.a. | 2.23583 |
| 138 | chr19 | 56538464 | hetero | NLRP5 | nonsynonymous SNV | NM_153447 | exon7 | c.G865A | p.G289R | not tolerated | probably damaging | 1.000 (sensitivity: 0.00; specificity: 1.00) | 0.971024 |
| 897 | chr7 | 156752722 | hetero | NOM1 | nonsynonymous SNV | NM_138400 | exon4 | c.G1486C | p.E496Q | not tolerated | probably damaging | 0.999 (sensitivity: 0.14; specificity: 0.99) | 2.283 |
| 138 | chr11 | 63994387 | hetero | NUDT22 | nonsynonymous SNV | NM_032344 | exon2 | c.G263A | p.R88Q | not tolerated | possibly damaging | 0.698 (sensitivity: 0.86; specificity: 0.92) | 0.0143937 |
| 138 | chr8 | 101733670 | hetero | PABPC1 | nonsynonymous SNV | NM_002568 | exon1 | c.A142C | p.T48P | not tolerated | probably damaging | 1.000 (sensitivity: 0.00; specificity: 1.00) | 1.52072 |
| 138 | chr9 | 128678097 | hetero | PBX3 | nonsynonymous SNV | NM_001134778 | exon3 | c.C182T | p.A61V | not tolerated | possibly damaging | 0.948 (sensitivity: 0.79; specificity: 0.95) | 2.941 |
| 138 | chr11 | 14666027 | hetero | PDE3B | nonsynonymous SNV | NM_000922 | exon1 | c.C406T | p.L136F | not tolerated | possibly damaging | 0.931 (sensitivity: 0.81; specificity: 0.94) | 0.103031 |
| 138 | chr1 | 144923728 | hetero | PDE4DIP | frameshift deletion | NM_001198834 | exon6 | c.729delA | p.E243fs | n.a. | n.a. | n.a. | n.a. |
| 138 | chr16 | 88786879 | hetero | PIEZO1 | nonsynonymous SNV | NM_001142864 | exon41 | c.C5863T | p.R1955C | not tolerated | probably damaging | 0.991 (sensitivity: 0.71; specificity: 0.97) | 2.55568 |
| 897 | chr12 | 133209362 | hetero | POLE | frameshift deletion | NM_006231 | exon44 | c.6023delA | p.Y2008fs | n.a. | n.a. | n.a. | n.a. |
| 897 | chr14 | 63848780 | hetero | PPP2R5E | nonsynonymous SNV | NM_006246 | exon13 | c.G1298A | p.R433H | not tolerated | probably damaging | 0.996 (sensitivity: 0.55; specificity: 0.98) | 2.709 |
| 138 | chr20 | 62195457 | hetero | PRIC285 | nonsynonymous SNV | NM_033405 | exon3 | c.G3011A | p.R1004Q | not tolerated | possibly damaging | 0.951 (sensitivity: 0.79; specificity: 0.95) | 0.658969 |
| 138 | chr2 | 37513341 | hetero | PRKD3 | nonsynonymous SNV | NM_005813 | exon5 | c.C889T | p.R297C | not tolerated | probably damaging | 1.000 (sensitivity: 0.00; specificity: 1.00) | 2.709 |
| 138 | chr17 | 27031323 | hetero | PROCA1 | splicing | NM_152465 |  | c.356+2T>G |  | n.a. | n.a. | n.a. | 1.96994 |
| 897 | chr5 | 120022381 | hetero | PRR16 | nonsynonymous SNV | NM_016644 | exon3 | c.A823C | p.K275Q | not tolerated | robably damaging | 1.000 (sensitivity: 0.00; specificity: 1.00) | 1.99492 |
| 138 | chr19 | 844017 | hetero | PRTN3 | nonsynonymous SNV | NM_002777 | exon3 | c.G352A | p.D118N | not tolerated | probably damaging | 1.000 (sensitivity: 0.00; specificity: 1.00) | 1.50752 |
| 138 | chr19 | 43570667 | hetero | PSG2 | splicing | NM_031246 |  | c.1048+2T>G |  | n.a. | n.a. | n.a. | 0.664063 |
| 897 | chr5 | 38945757 | hetero | RICTOR | nonsynonymous SNV | NM_152756 | exon34 | c.C4469T | p.T1490M | not tolerated | probably damaging | 0.999 (sensitivity: 0.14; specificity: 0.99) | 2.882 |
| 138 | chr16 | 2004119 | homo | RPL3L | nonsynonymous SNV | NM_005061 | exon2 | c.G34A | p.G12R | not tolerated | probably damaging | 1.000 (sensitivity: 0.00; specificity: 1.00) | 2.39225 |
| 138 | chr19 | 38948174 | hetero | RYR1 | nonsynonymous SNV | NM_001042723 | exon17 | c.G1829A | p.G610D | not tolerated | probably damaging | 1.000 (sensitivity: 0.00; specificity: 1.00) | 2.07554 |
| 897 | chr19 | 38945887 | hetero | RYR1 | nonsynonymous SNV | NM_000540 | exon14 | c.A1453G | p.M485V | not tolerated | benign | 0.069 (sensitivity: 0.94; specificity: 0.84) | -0.212016 |
| 138 | chr19 | 1122146 | hetero | SBNO2 | nonsynonymous SNV | NM_014963 | exon11 | c.G1141C | p.E381Q | not tolerated | possibly damaging | 0.793 (sensitivity: 0.84; specificity: 0.93) | 1.939 |
| 897 | chr2 | 110065896 | hetero | SH3RF3 | nonsynonymous SNV | NM_001099289 | exon8 | c.C2099T | p.S700F | not tolerated | probably damaging | 0.966 (sensitivity: 0.78; specificity: 0.95) | 0.61663 |
| 138 | chr17 | 18259294 | hetero | SHMT1 | nonsynonymous SNV | NM_148918 | exon2 | c.T2A | p.M1K | not tolerated | possibly damaging | 0.932 (sensitivity: 0.80; specificity: 0.94) | 2.02202 |
| 897 | chr13 | 103718287 | hetero | SLC10A2 | nonsynonymous SNV | NM_000452 | exon1 | c.T313C | p.C105R | not tolerated | probably damaging | 1.000 (sensitivity: 0.00; specificity: 1.00) | 1.96698 |
| 897 | chr17 | 78210808 | hetero | SLC26A11 | frameshift deletion | NM_001166349 | exon7 | c.819delA | p.T273fs | n.a. | n.a. | n.a. | n.a. |
| 897 | chr19 | 17988620 | hetero | SLC5A5 | stopgain SNV | NM_000453 | exon6 | c.C787T | p.Q263X | n.a. | n.a. | n.a. | 2.675 |
| 897 | chr16 | 3658540 | hetero | SLX4 | frameshift insertion | NM_032444 | exon2 | c.425_426insG | p.G142fs | n.a. | n.a. | n.a. | n.a. |
| 897 | chr11 | 57314091 | hetero | SMTNL1 | nonsynonymous SNV | NM_001105565 | exon6 | c.C1306T | p.R436W | not tolerated | probably damaging | 1.000 (sensitivity: 0.00; specificity: 1.00) | 0.103811 |
| 138 | chr20 | 48523066 | hetero | SPATA2 | nonsynonymous SNV | NM_001135773 | exon3 | c.G653A | p.R218Q | not tolerated | probably damaging | 0.975 (sensitivity: 0.76; specificity: 0.96) | 2.674 |
| 138 | chr4 | 168155741 | hetero | SPOCK3 | splicing | NM_001204352 |  | c.1-366G>C |  | n.a. | n.a. | n.a. | 0.609339 |
| 138 | chr5 | 171484476 | hetero | STK10 | splicing | NM_005990 | exon15 | c.C2214T | p.D738D | n.a. | n.a. | n.a. | 0.209504 |
| 897 | chr19 | 47226142 | hetero | STRN4 | nonsynonymous SNV | NM_001039877 | exon14 | c.C1852T | p.R618C | not tolerated | probably damaging | 0.993 (sensitivity: 0.70; specificity: 0.97) | 1.32417 |
| 138 | chr6 | 144507760 | hetero | STX11 | splicing | NM_003764 |  | c.1-5G>A |  | n.a. | n.a. | n.a. | 0.756701 |
| 897 | chr11 | 64882868 | hetero | TM7SF2 | splicing | NM_003273 |  | c.973+2T>G |  | n.a. | n.a. | n.a. | 2.02943 |
| 897 | chr3 | 36896793 | hetero | TRANK1 | nonsynonymous SNV | NM_014831 | exon12 | c.G4288A | p.V1430M | not tolerated | probably damaging | 1.000 (sensitivity: 0.00; specificity: 1.00) | 2.78 |
| 897 | chr5 | 114513491 | hetero | TRIM36 | nonsynonymous SNV | NM_001017398 | exon2 | c.G142A | p.E48K | not tolerated | possibly damaging | 0.884 (sensitivity: 0.82; specificity: 0.94) | 0.316087 |
| 138 | chr21 | 38560797 | hetero | TTC3 | nonsynonymous SNV | NM_001001894 | exon39 | c.T4925G | p.V1642G | n.a. | n.a. | n.a. | 1.98714 |
| 138 | chr21 | 43535988 | hetero | UMODL1 | nonsynonymous SNV | NM_001199528 | exon14 | c.C2152T | p.R718W | not tolerated | probably damaging | 0.987 (sensitivity: 0.73; specificity: 0.96) | 1.07652 |
| 897 | chr7 | 48147917 | hetero | UPP1 | nonsynonymous SNV | NM_003364 | exon9 | c.T896G | p.V299G | not tolerated | probably damaging | 0.999 (sensitivity: 0.14; specificity: 0.99) | 1.98703 |
| 897 | chr8 | 11995785 | hetero | USP17L2 | nonsynonymous SNV | NM_201402 | exon1 | c.A485T | p.H162L | not tolerated | probably damaging | 0.999 (sensitivity: 0.14; specificity: 0.99) | 0.605181 |
| 897 | chr8 | 11995540 | hetero | USP17L2 | nonsynonymous SNV | NM_201402 | exon1 | c.G730A | p.E244K | not tolerated | possibly damaging | 0.812 (sensitivity: 0.84; specificity: 0.93) | 0.70674 |
| 897 | chr12 | 118511544 | hetero | VSIG10 | frameshift deletion | NM_019086 | exon5 | c.1178delC | p.P393fs | n.a. | n.a. | n.a. | n.a. |
| 138 | chr14 | 100826957 | hetero | WARS | nonsynonymous SNV | NM_004184 | exon4 | c.G356A | p.R119Q | not tolerated | probably damaging | 0.999 (sensitivity: 0.14; specificity: 0.99) | 1.25958 |
| 897 | chr1 | 85598679 | homo | WDR63 | frameshift insertion | NM_145172 | exon23 | c.2675_2676insA | p.X892delinsX | n.a. | n.a. | n.a. | n.a. |
| 897 | chr5 | 112929029 | hetero | YTHDC2 | nonsynonymous SNV | NM_022828 | exon29 | c.G4242C | p.L1414F | not tolerated | probably damaging | 0.998 (sensitivity: 0.27; specificity: 0.99) | 1.54278 |
| 897 | chr15 | 41105019 | hetero | ZFYVE19 | nonsynonymous SNV | NM_001077268 | exon7 | c.C949T | p.R317W | not tolerated | probably damaging | 0.999 (sensitivity: 0.14; specificity: 0.99) | 2.41431 |
| 897 | chr19 | 37975192 | hetero | ZNF570 | nonsynonymous SNV | NM_144694 | exon5 | c.G668A | p.C223Y | not tolerated | probably damaging | 1.000 (sensitivity: 0.00; specificity: 1.00) | 2.37154 |
| 897 | chr19 | 53669013 | hetero | ZNF665 | nonsynonymous SNV | NM_024733 | exon4 | c.A730T | p.S244C | not tolerated | possibly damaging | 0.916 (sensitivity: 0.81; specificity: 0.94) | 0.124976 |
| 138 | chr16 | 30566897 | hetero | ZNF764 | nonsynonymous SNV | NM_033410 | exon3 | c.G845T | p.S282I | not tolerated | probably damaging | 0.999 (sensitivity: 0.14; specificity: 0.99) | 2.709 |
| 897 | chr19 | 22940192 | hetero | ZNF99 | frameshift insertion | NM_001080409 | exon4 | c.2518_2519insC | p.M840fs | n.a. | n.a. | n.a. | n.a. |

Note: chr=chromosome; n.a.=not applicable; hetero=heterozigous; homo=homozigous; SNV=signle nucleotide variation; n.d.=not determinable

Table S2. Variations predicted to impair protein function exclusive to Z-RTT patients

| sample | chr | genomic position | genotype | gene | variation type | RefSeq | exon | nucleotide variation | protein variation | SIFT | Polyphen | | PhyloP |
| --- | --- | --- | --- | --- | --- | --- | --- | --- | --- | --- | --- | --- | --- |
| 139 | chr11 | 130275887 | hetero | ADAMTS8 | nonsynonymous SNV | NM_007037 | exon9 | c.G2236A | p.G746S | not tolerated | probably damaging | 0.998 (sensitivity: 0.27; specificity: 0.99) | 2.47198 |
| 139 | chr10 | 61802478 | hetero | ANK3 | nonsynonymous SNV | NM_001204403 | exon43 | c.C5557T | p.R1853W | not tolerated | probably damaging | 0.997 (sensitivity: 0.41; specificity: 0.98) | 2.7004 |
| 139 | chr4 | 79512731 | hetero | ANXA3 | nonsynonymous SNV | NM_005139 | exon7 | c.C437T | p.S146F | not tolerated | probably damaging | 0.998 (sensitivity: 0.27; specificity: 0.99) | 2.52586 |
| 896 | chr8 | 37963081 | homo | ASH2L | nonsynonymous SNV | NM_004674 | exon1 | c.G13C | p.G5R | not tolerated | probably damaging | 1.000 (sensitivity: 0.00; specificity: 1.00) | 2.56956 |
| 896 | chr7 | 65553847 | hetero | ASL | nonsynonymous SNV | NM_001024946 | exon9 | c.G694A | p.E232K | not tolerated | probably damaging | 0.998 (sensitivity: 0.27; specificity: 0.99) | 2.756 |
| 139 | chr15 | 25963545 | hetero | ATP10A | splicing | NM_024490 | exon 8 | c.G1365T | p:A455A | n.a. | n.a. | n.a. | -0.0784567 |
| 139 | chr20 | 52570152 | hetero | BCAS1 | nonsynonymous SNV | NM_003657 | exon11 | c.C1499T | p.S500L | not tolerated | probably damaging | 0.995 (sensitivity: 0.68; specificity: 0.97) | 0.435843 |
| 139 | chr14 | 23467791 | hetero | C14orf93 | nonsynonymous SNV | NM_021944 | exon2 | c.G442A | p.V148M | not tolerated | probably damaging | 0.998 (sensitivity: 0.27; specificity: 0.99) | 2.71485 |
| 139 | chr6 | 26406283 | hetero | BTN3A1 | nonsynonymous SNV | NM_001145008 | exon3 | c.G232A | p.V78M | not tolerated | probably damaging | 0.995 (sensitivity: 0.68; specificity: 0.97) | 0.519701 |
| 896 | chr19 | 16623876 | hetero | C19orf44 | nonsynonymous SNV | NM_032207 | exon6 | c.C1691T | p.P564L | not tolerated | probably damaging | 1.000 (sensitivity: 0.00; specificity: 1.00) | 2.461 |
| 139 | chr18 | 66721271 | homo | CCDC102B | nonsynonymous SNV | NM_024781 | exon8 | c.A1439T | p.D480V | not tolerated | probably damaging | 0.971 (sensitivity: 0.77; specificity: 0.96) | 0.218362 |
| 139 | chr10 | 12940648 | hetero | CCDC3 | nonsynonymous SNV | NM_031455 | exon3 | c.T581C | p.F194S | not tolerated | probably damaging | 0.963 (sensitivity: 0.78; specificity: 0.95) | 2.01744 |
| 139 | chr17 | 40832326 | hetero | CCR10 | nonsynonymous SNV | NM_016602 | exon2 | c.A334C | p.T112P | not tolerated | possibly damaging | 0.891 (sensitivity: 0.82; specificity: 0.94) | 1.58876 |
| 139 | chr1 | 146759412 | hetero | CHD1L | nonsynonymous SNV | NM_004284 | exon19 | c.G2320A | p.D774N | not tolerated | probably damaging | 1.000 (sensitivity: 0.00; specificity: 1.00) | 2.6 |
| 139 | chr19 | 45572384 | hetero | CLASRP | splicing | NM_007056 | exon17 | c.1827+2T>G |  | n.a. | n.a. | n.a. | 0.995291 |
| 139 | chr16 | 23400270 | hetero | COG7 | nonsynonymous SNV | NM_153603 | exon17 | c.G2284A | p.V762M | not tolerated | probably damaging | 0.979 (sensitivity: 0.76; specificity: 0.96) | 0.526 |
| 139 | chr9 | 137702117 | hetero | COL5A1 | nonsynonymous SNV | NM_000093 | exon44 | c.C3491T | p.P1164L | not tolerated | probably damaging | 0.994 (sensitivity: 0.69; specificity: 0.97) | 2,00919 |
| 139 | chr3 | 98300354 | hetero | CPOX | nonsynonymous SNV | NM_000097 | exon6 | c.T1174G | p.Y392D | not tolerated | probably damaging | 1.000 (sensitivity: 0.00; specificity: 1.00) | 2.076 |
| 139 | chr4 | 155241663 | hetero | DCHS2 | nonsynonymous SNV | NM_017639 | exon14 | c.G3523A | p.E1175K | not tolerated | probably damaging | 1.000 (sensitivity: 0.00; specificity: 1.00) | 2.56248 |
| 139 | chr14 | 60616070 | hetero | DHRS7 | splicing | NM_016029 | exon7 | c.972+1G>A |  | n.a. | n.a. | n.a. | 2.66415 |
| 896 | chr12 | 124402341 | hetero | DNAH10 | nonsynonymous SNV | NM_207437 | exon63 | c.C10859T | p.T3620I | not tolerated | probably damaging | 0.999 (sensitivity: 0.14; specificity: 0.99) | 2.32203 |
| 139 | chr1 | 36306879 | hetero | EIF2C4 | nonsynonymous SNV | NM_017629 | exon14 | c.A1838C | p.H613P | not tolerated | probably damaging | 0.997 (sensitivity: 0.41; specificity: 0.98) | 2.29785 |
| 896 | chr17 | 56277684 | hetero | EPX | nonsynonymous SNV | NM_000502 | exon10 | c.C1636T | p.R546W | not tolerated | probably damaging | 0.989 (sensitivity: 0.72; specificity: 0.97) | -0.140598 |
| 139 | chr11 | 46748178 | hetero | F2 | splicing | NM_000506 | exon8 | c.1003+2T>G |  | n.a. | n.a. | n.a. | 1.82488 |
| 139 | chr14 | 75538258 | hetero | FAM164C | frameshift insertion | NM_024643 | exon2 | c.983_984insG | p.R328fs | n.a. | n.a. | n.a. | n.a. |
| 139 | chr3 | 43074779 | hetero | FAM198A | nonsynonymous SNV | NM_001129908 | exon2 | c.C1024T | p.R342W | not tolerated | probably damaging | 1.000 (sensitivity: 0.00; specificity: 1.00 | 0.645913 |
| 139 | chr12 | 100042182 | hetero | FAM71C | frameshift insertion | NM_153364 | exon1 | c.231_232insC | p.S77fs | n.a. | n.a. | n.a. | n.a. |
| 896 | chr19 | 5831525 | hetero | FUT6 | nonsynonymous SNV | NM_001040701 | exon2 | c.C1054T | p.R352C | not tolerated | probably damaging | 0.996 (sensitivity: 0.55; specificity: 0.98) | -0.159906 |
| 139 | chr5 | 153783664 | hetero | GALNT10 | nonsynonymous SNV | NM_198321 | exon8 | c.G1057T | p.V353L | n.a. | n.a. | n.a. | 2.533 |
| 139 | chr9 | 6554754 | hetero | GLDC | nonsynonymous SNV | NM_000170 | exon19 | c.G2230A | p.G744R | not tolerated | probably damaging | 1.000 (sensitivity: 0.00; specificity: 1.00) | 2.677 |
| 139 | chr10 | 121203194 | hetero | GRK5 | nonsynonymous SNV | NM_005308 | exon12 | c.G1196A | p.R399H | not tolerated | probably damaging | 0.998 (sensitivity: 0.27; specificity: 0.99) | 2.29594 |
| 896 | chr13 | 114018214 | hetero | GRTP1 | nonsynonymous SNV | NM_024719 | exon2 | c.A44G | p.Y15C | not tolerated | probably damaging | 1.000 (sensitivity: 0.00; specificity: 1.00) | 1.66253 |
| 139 | chr12 | 42512938 | hetero | GXYLT1 | nonsynonymous SNV | NM_001099650 | exon2 | c.A257T | p.H86L | not tolerated | possibly damaging | 0.937 (sensitivity: 0.80; specificity: 0.94) | 4.2512938 |
| 896 | chr1 | 9305172 | hetero | H6PD | nonsynonymous SNV | NM_004285 | exon2 | c.T179G | p.F60C | not tolerated | probably damaging | 1.000 (sensitivity: 0.00; specificity: 1.00) | 2.08687 |
| 139 | chr16 | 30006689 | hetero | HIRIP3 | nonsynonymous SNV | NM_003609 | exon2 | c.T161G | p.V54G | not tolerated | probably damaging | 0.995 (sensitivity: 0.68; specificity: 0.97) | 0.975567 |
| 139 | chr7 | 154876006 | hetero | HTR5A | nonsynonymous SNV | NM_024012 | exon2 | c.G883A | p.V295M | not tolerated | probably damaging | 0.989 (sensitivity: 0.72; specificity: 0.97) | 2.22085 |
| 896 | chr14 | 23845069 | hetero | IL25 | nonsynonymous SNV | NM_022789 | exon2 | c.C514T | p.R172W | not tolerated | probably damaging | 1.000 (sensitivity: 0.00; specificity: 1.00) | 0.105567 |
| 896 | chr1 | 24486098 | hetero | IL28RA | frameshift insertion | NM_173064 | exon5 | c.535_536insCATG | p.G179fs | n.a. | n.a. | n.a. | n.a. |
| 896 | chr12 | 121880638 | hetero | KDM2B | nonsynonymous SNV | NM_032590 | exon19 | c.G2606T | p.R869L | n.a. | n.a. | n.a. | 0.831504 |
| 139 | chr13 | 46942949 | hetero | KIAA0226L | splicing | NM_025113 | exon4 | c.T537G | p.G179G | n.a. | n.a. | n.a. | 2.33643 |
| 139 | chr1 | 900560 | hetero | KLHL17 | nonsynonymous SNV | NM_198317 | exon12 | c.A1918C | p.T640P | not tolerated | probably damaging | 0.995 (sensitivity: 0.68; specificity: 0.97) | 2.14883 |
| 896 | chr1 | 152733723 | hetero | KPRP | frameshift insertion | NM_001025231 | exon2 | c.1660_1661insC | p.R554fs | n.a. | n.a. | n.a. | n.a. |
| 139 | chr4 | 129012213 | hetero | LARP1B | nonsynonymous SNV | NM_018078 | exon6 | c.G416A | p.S139N | not tolerated | probably damaging | 0.995 (sensitivity: 0.68; specificity: 0.97) | 2.407 |
| 139 | chr9 | 139649782 | hetero | LCN8 | nonsynonymous SNV | NM_178469 | exon5 | c.G335A | p.R112Q | not tolerated | probably damaging | 1.000 (sensitivity: 0.00; specificity: 1.00) | 1.85978 |
| 896 | chr11 | 551822 | hetero | LRRC56 | nonsynonymous SNV | NM_198075 | exon10 | c.C968T | p.T323I | not tolerated | probably damaging | 1.000 (sensitivity: 0.00; specificity: 1.00) | 1.03995 |
| 139 | chr11 | 71818990 | hetero | LRTOMT | nonsynonymous SNV | NM_001145308 | exon6 | c.T380G | p.V127G | not tolerated | probably damaging | 0.966 (sensitivity: 0.78; specificity: 0.95) | 2.14238 |
| 896 | chr11 | 58979668 | hetero | MPEG1 | nonsynonymous SNV | NM_001039396 | exon1 | c.C671T | p.S224F | not tolerated | probably damaging | 0.997 (sensitivity: 0.41; specificity: 0.98) | 2.42688 |
| 896 | chr1 | 16916395 | hetero | NBPF1 | nonsynonymous SNV | NM_17940 | exon8 | c.A277T | p.R93W | not tolerated | n.d. | n.d. | 0.448354 |
| 139 | chr7 | 158494629 | hetero | NCAPG2 | splicing | NM_017760 | intron | c.IVS1-39G>A |  | n.a. | n.a. | n.a. | 0.499449 |
| 139 | chr5 | 140795208 | hetero | PCDHGA10 | frameshift deletion | NM_032090 | exon1 | c.2467delA | p.K823fs | n.a. | n.a. | n.a. | n.a. |
| 139 | chr9 | 78711018 | hetero | PCSK5 | splicing | NM_006200 | exon8 | c.C1107T | p.I369I | n.a. | n.a. | n.a. | -0.181528 |
| 139 | chr12 | 41587882 | hetero | PDZRN4 | splicing | NM_001164595 | exon3 | c.736-1G>C |  | n.a. | n.a. | n.a. | 1.22374 |
| 139 | chr15 | 42434285 | hetero | PLA2G4F | nonsynonymous SNV | NM_213600 | exon20 | c.T2447G | p.V816G | not tolerated | probably damaging | 0.999 (sensitivity: 0.14; specificity: 0.99) | 2.05502 |
| 139 | chr1 | 12939546 | hetero | PRAMEF4 | frameshift insertion | NM_001009611 | exon4 | c.1255_1256insC | p.R419fs | n.a. | n.a. | n.a. | n.a. |
| 896 | chr19 | 38872779 | homo | PSMD8 | nonsynonymous SNV | NM_002812 | exon6 | c.G826C | p.E276Q | not tolerated | probably damaging | 0.999 (sensitivity: 0.14; specificity: 0.99) | 1.25839 |
| 139 | chr6 | 31106500 | hetero | PSORS1C1 | frameshift insertion | NM_014068 | exon5 | c.112_113insC | p.P38fs | n.a. | n.a. | n.a. | n.a. |
| 896 | chr7 | 100961427 | hetero | RABL5 | nonsynonymous SNV | NM_022777 | exon3 | c.T184C | p.W62R | not tolerated | probably damaging | 0.999 (sensitivity: 0.14; specificity: 0.99) | 1.08191 |
| 139 | chr10 | 112544125 | hetero | RBM20 | nonsynonymous SNV | NM_001134363 | exon4 | c.C1364T | p.S455L | not tolerated | possibly damaging | 0.614 (sensitivity: 0.87; specificity: 0.91) | 2.5633 |
| 896 | chr21 | 43905850 | hetero | RSPH1 | nonsynonymous SNV | NM_080860 | exon5 | c.G430A | p.G144R | not tolerated | probably damaging | 1.000 (sensitivity: 0.00; specificity: 1.00) | 2.5 |
| 139 | chr6 | 130505271 | hetero | SAMD3 | nonsynonymous SNV | NM_001017373 | exon7 | c.C631G | p.L211V | not tolerated | probably damaging | 0.999 (sensitivity: 0.14; specificity: 0.99) | 0.679654 |
| 139 | chr10 | 102743493 | hetero | SEMA4G | nonsynonymous SNV | NM_017893 | exon14 | c.C2137T | p.R713C | not tolerated | possibly damaging | 0.956 (sensitivity: 0.79; specificity: 0.95) | 0.576567 |
| 896 | chr14 | 94756503 | hetero | SERPINA10 | nonsynonymous SNV | NM_016186 | exon2 | c.C428A | p.S143Y | not tolerated | possibly damaging | 0.563 (sensitivity: 0.88; specificity: 0.91) | 0.427181 |
| 896 | chr7 | 37953841 | hetero | SFRP4 | nonsynonymous SNV | NM_003014 | exon3 | c.C566T | p.T189M | not tolerated | probably damaging | 1.000 (sensitivity: 0.00; specificity: 1.00) | 1.53587 |
| 896 | chr1 | 36772294 | homo | SH3D21 | nonsynonymous SNV | NM_001162530 | exon2 | c.C100T | p.R34W | not tolerated | probably damaging | 1.000 (sensitivity: 0.00; specificity: 1.00) | 0.382559 |
| 896 | chr11 | 130785638 | hetero | SNX19 | nonsynonymous SNV | NM_014758 | exon1 | c.G197A | p.G66D | not tolerated | probably damaging | 1.000 (sensitivity: 0.00; specificity: 1.00) | 1.20113 |
| 896 | chr15 | 42160366 | hetero | SPTBN5 | frameshift deletion | NM_016642 | exon34 | c.6001_6002delCA | p.2001_2001del | n.a. | n.a. | n.a. | n.a. |
| 139 | chr5 | 79617198 | hetero | SPZ1 | frameshift insertion | NM_032567 | exon1 | c.1165_1166insA | p.K389fs | n.a. | n.a. | n.a. | n.a. |
| 139 | chr4 | 76452302 | hetero | THAP6 | nonsynonymous SNV | NM_144721 | exon5 | c.T547A | p.L183M | not tolerated | probably damaging | 0.999 (sensitivity: 0.14; specificity: 0.99) | -0.666157 |
| 139 | chr18 | 214519 | hetero | THOC1 | splicing | NM_005131 | intron | c.106_107insCAA |  | n.a. | n.a. | n.a. | 0.516097 |
| 139 | chr9 | 71831325 | hetero | TJP2 | nonsynonymous SNV | NM_001170415 | exon3 | c.C197T | p.T66M | not tolerated | probably damaging | 1.000 (sensitivity: 0.00; specificity: 1.00) | 2.805 |
| 139 | chr6 | 30314508 | hetero | TRIM39-RPP21 | nonsynonymous SNV | NM_001199119 | exon10 | c.C1433T | p.P478L | not tolerated | possibly damaging | 0.584 (sensitivity: 0.88; specificity: 0.91) | 2.23 |
| 139 | chr15 | 31354782 | hetero | TRPM1 | splicing | NM_001252024 | exon9 | c.C1089T | p.L363L | n.a. | n.a. | n.a. | -1.5554 |
| 139 | chr1 | 156553620 | hetero | TTC24 | nonsynonymous SNV | NM_001105669 | exon6 | c.T1184G | p.V395G | not tolerated | probably damaging | 1.000 (sensitivity: 0.00; specificity: 1.00) | 2.14802 |
| 139 | chr4 | 69962882 | hetero | UGT2B7 | nonsynonymous SNV | NM_001074 | exon1 | c.T644A | p.I215N | not tolerated | possibly damaging | 0.723 (sensitivity: 0.86; specificity: 0.92) | 1.098 |
| 139 | chr4 | 177058733 | hetero | WDR17 | nonsynonymous SNV | NM_181265 | exon9 | c.A1330C | p.K444Q | not tolerated | possibly damaging | 0.822 (sensitivity: 0.84; specificity: 0.93) | 2.08933 |
| 139 | chr7 | 158705718 | hetero | WDR60 | nonsynonymous SNV | NM_018051 | exon13 | c.C1633G | p.Q545E | not tolerated | probably damaging | 1.000 (sensitivity: 0.00; specificity: 1.00) | 2.534 |
| 139 | chr3 | 39229879 | hetero | XIRP1 | nonsynonymous SNV | NM_194293 | exon2 | c.C1058T | p.A353V | not tolerated | probably damaging | 0.957 (sensitivity: 0.78; specificity: 0.95) | 2.40746 |
| 139 | chr7 | 129662189 | hetero | ZC3HC1 | frameshift deletion | NM_016478 | exon9 | c.1406_1409delTGTT | p.469_470del | n.a. | n.a. | n.a. | n.a. |
| 139 | chr11 | 57456075 | hetero | ZDHHC5 | nonsynonymous SNV | NM_015457 | exon4 | c.A322C | p.T108P | not tolerated | probably damaging | 1.000 (sensitivity: 0.00; specificity: 1.00) | 2.05976 |
| 139 | chr20 | 25657106 | hetero | ZNF337 | nonsynonymous SNV | NM_015655 | exon5 | c.A818G | p.Y273C | not tolerated | probably damaging | 0.991 (sensitivity: 0.71; specificity: 0.97) | 0.899772 |

Note: chr=chromosome; n.a.=not applicable; hetero=heterozigous; homo=homozigous; SNV=signle nucleotide variation; n.d.=not determinable

**Table S3. Variations predicted to impair protein function in discordant RTT patients.**

| sample | chr | genomic position | genotype | gene | variation type | RefSeq | exon | nucleotide variation | protein variation | SIFT | PolyPhen2 | | PhyloP |
| --- | --- | --- | --- | --- | --- | --- | --- | --- | --- | --- | --- | --- | --- |
| 138;139 | chr17 | 66925293 | homo | ABCA8 | stopgain SNV | NM_007168 | exon8 | c.G1022A | p.W341X | n.a. | n.a. |  | 2.54809 |
| 138;139 | chr12 | 109690847 | hetero | ACACB | nonsynonymous SNV | NM_001093 | exon42 | c.A5929C | p.N1977H | not tolerated | probably damaging | 1.000 (sensitivity: 0; specificity: 1) | 2.32163 |
| 138;139 | chr15 | 84611386 | hetero | ADAMTSL3 | nonsynonymous SNV | NM_207517 | exon18 | c.G2156A | p.C719Y | not tolerated | probably damaging | 1.000 (sensitivity: 0.00; specificity: 1.00) | 2.683 |
| 138;139 | chr16 | 4015987 | hetero | ADCY9 | frameshift insertion | NM_001116 | exon11 | c.3850_3851insT | p.S1284fs | n.a. | n.a. |  | n.a. |
| 138;139 | chr2 | 73675227 | hetero | ALMS1 | nonframeshift insertion | NM_015120 | exon8 | c.1571_1572insCTC | p.S524delinsSS | n.a. | n.a. |  | 0.515816 |
| 138;139;897 | chr18 | 43833701 | hetero | C18orf25 | nonframeshift insertion | NM_001008239 | exon3 | c.755_756insCTG | p.S252delinsSC | n.a. | n.a. |  | 0.594874 |
| 138;139 | chr6 | 48036051 | hetero | C6orf138 | nonsynonymous SNV | NM_207499 | exon1 | c.T341G | p.V114G | not tolerated | possibly damaging | 0.567 (sensitivity: 0.88; specificity: 0.91) | 1.90581 |
| 138;139 | chr8 | 10532226 | hetero | C8orf74 | nonsynonymous SNV | NM_001040032 | exon2 | c.G119A | p.R40Q | not tolerated | probably damaging | 1.000 (sensitivity: 0.00; specificity: 1.00) | 2.348 |
| 896;897 | chr2 | 132290912 | hetero | CCDC74A | nonsynonymous SNV | NM_138770 | exon8 | c.G1078A | p.E360K | not tolerated | probably damaging | 0.999 (sensitivity: 0.14; specificity: 0.99) | 1.41485 |
| 139;897 | chr1 | 54605318 | hetero | CDCP2 | frameshift insertion | NM_201546 | exon4 | c.1224_1225insCC | p.P408fs | n.a. | n.a. |  | n.a. |
| 139;897 | chr12 | 51723598 | hetero | CELA1 | frameshift insertion | NM_001971 | exon7 | c.628_629insC | p.L210fs | n.a. | n.a. |  | n.a. |
| 138;139 | chr15 | 90774213 | hetero | CIB1 | nonsynonymous SNV | NM_006384 | exon6 | c.C507A | p.N169K | not tolerated | probably damaging | 1.000 (sensitivity: 0.00; specificity: 1.00) | -0.634189 |
| 138;139 | chr16 | 70543283 | hetero | COG4 | nonsynonymous SNV | NM_015386 | exon7 | c.C853T | p.R285C | not tolerated | probably damaging | 1.000 (sensitivity: 0.00; specificity: 1.00) | 1.38175 |
| 896;897 | chr2 | 38298188 | hetero | CYP1B1 | nonsynonymous SNV | NM_000104 | exon3 | c.C1309G | p.P437A | not tolerated | probably damaging | 1.000 (sensitivity: 0.00; specificity: 1.00) | 2.824 |
| 896;897 | chr4 | 955531 | hetero | DGKQ | nonsynonymous SNV | NM_001347 | exon20 | c.C2407T | p.R803W | not tolerated | probably damaging | 0.986 (sensitivity: 0.74; specificity: 0.96) | 2.44252 |
| 138;139;896 | chr19 | 49442849 | hetero | DHDH | frameshift insertion | NM_014475 | exon4 | c.511_512insG | p.G171fs | n.a. | n.a. |  | n.a. |
| 896;897 | chr21 | 37617750 | hetero | DOPEY2 | nonsynonymous SNV | NM_005128 | exon19 | c.C3472T | p.R1158C | not tolerated | problaby damaging | 0.997 (sensitivity: 0.41; specificity: 0.98) | 2.5111 |
| 138;139;897 | chr11 | 125452300 | homo | EI24 | frameshift insertion | NM_001007277 | exon9 | c.733_734insC | p.P245fs | n.a. | n.a. |  | n.a. |
| 139;897 | chr6 | 71187020 | hetero | FAM135A | nonsynonymous SNV | NM_020819 | exon6 | c.A398C | p.H133P | not tolerated | probably damaging | 1.000 (sensitivity: 0.00; specificity: 1.00) | 2.08374 |
| 138;139 | chr3 | 150387210 | hetero | FAM194A | frameshift deletion | NM_152394 | exon12 | c.1371delT | p.I457fs | n.a. | n.a. |  | n.a. |
| 896;897 | chr10 | 124608903 | hetero | FAM24B | nonsynonymous SNV | NM_152644 | exon4 | c.G145A | p.V49M | not tolerated | possibly damaging | 0.896 (sensitivity: 0.82; specificity: 0.94 | 0.0250394 |
| 896;897 | chr16 | 67577275 | hetero | FAM65A | nonsynonymous SNV | NM_001193524 | exon14 | c.C2545T | p.R849W | not tolerated | probably damaging | 1.000 (sensitivity: 0.00; specificity: 1.00) | 1.30967 |
| 138;139 | chr8 | 124989741 | hetero | FER1L6 | nonsynonymous SNV | NM_001039112 | exon10 | c.C955T | p.R319W | not tolerated | probably damaging | 1.000 (sensitivity: 0.00; specificity: 1.00) | 2.55114 |
| 896;897 | chr12 | 52215875 | hetero | FIGNL2 | nonsynonymous SNV | NM_001013690 | exon2 | c.T323C | p.L108S | not tolerated | possibly damaging | 0.952 (sensitivity: 0.79; specificity: 0.95) | 2.09686 |
| 138;139 | chr1 | 24192072 | hetero | FUCA1 | nonsynonymous SNV | NM_000147 | exon2 | c.T433C | p.W145R | not tolerated | probably damaging | 0.999 (sensitivity: 0.14; specificity: 0.99) | 2.08553 |
| 138;139;897 | chr1 | 156565049 | homo | GPATCH4 | frameshift insertion | NM_182679 | exon8 | c.1068_1069insGT | p.T356fs | n.a. | n.a. |  | n.a. |
| 138;139 | chr6 | 143094132 | hetero | HIVEP2 | nonsynonymous SNV | NM_006734 | exon5 | c.G1744A | p.V582M | not tolerated | probably damaging | 0.995 (sensitivity: 0.68; specificity: 0.97) | 2.55113 |
| 138;139 | chrX | 53283827 | hetero | IQSEC2 | nonsynonymous SNV | NM_001111125 | exon4 | c.G1286A | p.G429E | not tolerated | possibly damaging | 0.856 (sensitivity: 0.83; specificity: 0.93) | 2.08543 |
| 138;139 | chr12 | 53184619 | hetero | KRT3 | splicing | NM_057088 | exon9 | c.1570+2->T |  | n.a. | n.a. |  | n.a. |
| 138;139 | chr12 | 53169344 | hetero | KRT76 | nonsynonymous SNV | NM_015848 | exon2 | c.T643C | p.W215R | not tolerated | probably damaging | 1.000 (sensitivity: 0.00; specificity: 1.00) | 0.606724 |
| 139;896;897 | chr17 | 39254335 | homo | KRTAP4-8 | frameshift insertion | NM_031960 | exon1 | c.1_2insA | p.M1fs | n.a. | n.a. |  | n.a. |
| 138;139;897 | chr14 | 50750699 | hetero | L2HGDH | nonsynonymous SNV | NM_024884 | exon5 | c.T593G | p.V198G | not tolerated | probably damaging | 0.997 (sensitivity: 0.41; specificity: 0.98) | 2.24009 |
| 896;897 | chrX | 26212477 | hetero | MAGEB6 | nonsynonymous SNV | NM_173523 | exon2 | c.G514C | p.G172R | n.d. | possibly damaging | 0.911 (sensitivity: 0.81; specificity: 0.94) | -0.874701 |
| 138;139 | chr15 | 75658932 | hetero | MAN2C1 | nonsynonymous SNV | NM_006715 | exon4 | c.G353A | p.G118D | not tolerated | probably damaging | 0.987 (sensitivity: 0.73; specificity: 0.96) | 2.01891 |
| 138;139 | chr2 | 210594973 | hetero | MAP2 | nonsynonymous SNV | NM_031847 | exon13 | c.T1361C | p.I454T | not tolerated | possibly damaging | 0.835 (sensitivity: 0.84; specificity: 0.93) | 2.03455 |
| 138;896;897 | chr7 | 15725797 | hetero | MEOX2 | nonframeshift deletion | NM_005924 | exon1 | c.228_230delTGG | p.76_77del | n.a. | n.a. |  | 1.88188 |
| 138 | chr6 | 31380157 | homo | MICA | frameshift deletion | NM_001177519 | exon5 | c.949_952delGCTG | p.317_318del | n.a. | n.a. |  | n.a. |
| 139 | chr6 | 31380161 | hetero | MICA | frameshift insertion | NM_001177519 | exon5 | c.953_954insCT | p.G318fs | n.a. | n.a. |  | n.a. |
| 138;139 | chr7 | 151945071 | hetero | MLL3 | stopgain SNV | NM_170606 | exon14 | c.2447_2448insA | p.Y816_I817delinsX | n.a. | n.a. |  | 2.08441 |
| 138;139 | chr13 | 29608190 | hetero | MTUS2 | nonsynonymous SNV | NM_001033602 | exon2 | c.T2404C | p.S802P | n.d. | probably damaging | 0.996 (sensitivity: 0.55; specificity: 0.98) | 0.840433 |
| 138;139 | chr3 | 108175695 | hetero | MYH15 | nonsynonymous SNV | NM_014981 | exon20 | c.C2116G | p.R706G | not tolerated | possibly damaging | 0.825 (sensitivity: 0.84; specificity: 0.93) | -0.430354 |
| 139;897 | chr13 | 52718050 | hetero | NEK3 | frameshift insertion | NM_001146099 | exon10 | c.876_877insA | p.K292fs | n.a. | n.a. |  | n.a. |
| 138;139 | chr5 | 140166306 | hetero | PCDHA1 | nonsynonymous SNV | NM_031410 | exon1 | c.A431C | p.E144A | not tolerated | probably damging | 1.000 (sensitivity: 0.00; specificity: 1.00) | 1.62157 |
| 896;897 | chr1 | 233334738 | hetero | PCNXL2 | nonsynonymous SNV | NM_014801 | exon15 | c.C3013T | p.R1005W | not tolerated | probably damaging | 1.000 (sensitivity: 0.00; specificity: 1.00) | 1.22961 |
| 896;897 | chr18 | 10696470 | hetero | PIEZO2 | nonsynonymous SNV | NM_022068 | exon42 | c.G6556A | p.V2186M | tolerated | probably damaging | 1.000 (sensitivity: 0.00; specificity: 1.00) | 2.48989 |
| 896;897 | chrX | 114426193 | hetero | RBMXL3 | nonsynonymous SNV | NM_001145346 | exon1 | c.G2189A | p.R730Q | tolerated | possibly damaging | 0.535 (sensitivity: 0.88; specificity: 0.90) | 0.15163 |
| 138;139 | chr14 | 24808726 | homo | RIPK3 | nonsynonymous SNV | NM_006871 | exon2 | c.G98T | p.G33V | not tolerated | probably damaging | 1.000 (sensitivity: 0.00; specificity: 1.00) | 2.4848 |
| 138;139 | chr8 | 10480111 | hetero | RP1L1 | nonsynonymous SNV | NM_178857 | exon2 | c.G601A | p.G201R | not tolerated | probably damaging | 1.000 (sensitivity: 0.00; specificity: 1.00) | 2.44 |
| 138;139 | chr11 | 63527340 | hetero | RTN3 | splicing | NM_001265590 |  | c.*1668delA |  | n.a. | n.a. |  | n.a. |
| 138;139 | chr7 | 92734158 | hetero | SAMD9 | nonsynonymous SNV | NM_017654 | exon3 | c.A1253G | p.N418S | n.d. | probably damaging | 0.999 (sensitivity: 0.14; specificity: 0.99) | 0.704087 |
| 138;139 | chr8 | 8235093 | hetero | SGK223 | nonsynonymous SNV | NM_001080826 | exon2 | c.C826T | p.R276W | not tolerated | probably damaging | 0.988 (sensitivity: 0.73; specificity: 0.96) | -0.186417 |
| 896;897 | chr20 | 44980715 | hetero | SLC35C2 | nonsynonymous SNV | NM_015945 | exon8 | c.C812G | p.T271S | not tolerated | probably damaging | 0.999 (sensitivity: 0.14; specificity: 0.99) | 2.50066 |
| 139;896;897 | chr17 | 48626182 | hetero | SPATA20 | nonsynonymous SNV | NM_022827 | exon5 | c.A373C | p.T125P | not tolerated | probably damaging | 0.964 (sensitivity: 0.78; specificity: 0.95) | 0.643622 |
| 138;139 | chr5 | 139931628 | homo | SRA1 | frameshift insertion | NM_001035235 | exon3 | c.328_329insAC | p.V110fs | n.a. | n.a. |  | n.a. |
| 138;139 | chr11 | 85436785 | hetero | SYTL2 | nonsynonymous SNV | NM_206928 | exon1 | c.C715T | p.R239W | not tolerated | possibly damaging | 0.909 (sensitivity: 0.81; specificity: 0.94) | 0.163929 |
| 138;139 | chr1 | 179609132 | hetero | TDRD5 | nonsynonymous SNV | NM_001199085 | exon10 | c.A1679G | p.Y560C | not tolerated | probably damaging | 1.000 (sensitivity: 0.00; specificity: 1.00) | 2.1341 |
| 138;139 | chr8 | 133898884 | hetero | TG | nonsynonymous SNV | NM_003235 | exon9 | c.C1267T | p.R423C | not tolerated | possibly damaging | 0.876 (sensitivity: 0.83; specificity: 0.93) | 1.3699 |
| 138;139 | chr8 | 133885342 | hetero | TG | nonsynonymous SNV | NM_003235 | exon5 | c.C514T | p.L172F | not tolerated | probably damaging | 1.000 (sensitivity: 0.00; specificity: 1.00) | 2.768 |
| 138;139 | chr1 | 9667796 | hetero | TMEM201 | nonsynonymous SNV | NM_001130924 | exon7 | c.C1342T | p.R448C | not tolerated | probably damaging | 1.000 (sensitivity: 0.00; specificity: 1.00) | 2.5718 |
| 896;897 | chr12 | 51236806 | hetero | TMPRSS12 | stopgain SNV | NM_182559 | exon1 | c.C59G | p.S20X | n.a. | n.a. |  | -0.232496 |
| 139;897 | chr5 | 36036005 | hetero | UGT3A2 | nonsynonymous SNV | NM_001168316 | exon6 | c.T1265G | p.V422G | not tolerated | possibly damaging | 0.736 (sensitivity: 0.85; specificity: 0.92) | -0.0246614 |
| 138;139 | chr11 | 17552814 | hetero | USH1C | nonsynonymous SNV | NM_153676 | exon4 | c.C274T | p.R92C | not tolerated | probably damaging | 1.000 (sensitivity: 0.00; specificity: 1.00) | 2.688 |
| 896;897 | chr19 | 12460714 | hetero | ZNF442 | nonsynonymous SNV | NM_030824 | exon6 | c.A1685G | p.H562R | not tolerated | probably damaging | 0.984 (sensitivity: 0.74; specificity: 0.96) | 0.595961 |
| 139;897 | chr19 | 22817274 | hetero | ZNF492 | splicing | NM_020855 |  | c.-95delC |  | n.a. | n.a. |  | n.a. |
| 138;139;897 | chr19 | 20807177 | homo | ZNF626 | frameshift insertion | NM_001076675 | exon4 | c.1505_1506insT | p.I502fs | n.a. | n.a. |  | n.a. |

Note: chr=chromosome; n.a.=not applicable; hetero=heterozigous; homo=homozigous; SNV=signle nucleotide variation; n.d.=not determinable

**References S1**

1. Kanehisa M, Goto S (2000) KEGG: kyoto encyclopedia of genes and genomes. Nucleic Acids Res 28: 27-30.

2. Kanehisa M, Goto S, Sato Y, Furumichi M, Tanabe M (2012) KEGG for integration and interpretation of large-scale molecular data sets. Nucleic Acids Res 40: D109-14.

3. De Felice C, Signorini C, Leoncini S, Pecorelli A, Durand T*, et al.* (2012) The role of oxidative stress in Rett syndrome: an overview. Ann N Y Acad Sci 1259: 121-35.

4. De Felice C, Signorini C, Durand T, Ciccoli L, Leoncini S*, et al.* (2012) Partial rescue of Rett syndrome by omega-3 polyunsaturated fatty acids (PUFAs) oil. Genes Nutr 7: 447-58.

5. De Felice C, Ciccoli L, Leoncini S, Signorini C, Rossi M*, et al.* (2009) Systemic oxidative stress in classic Rett syndrome. Free Radic Biol Med 47: 440-8.

6. Pecorelli A, Ciccoli L, Signorini C, Leoncini S, Giardini A*, et al.* (2011) Increased levels of 4HNE-protein plasma adducts in Rett syndrome. Clin Biochem 44: 368-71.
